# Breast cancer induces CD62L^+^ Kupffer cells via DMBT1 to promote neutrophil extracellular trap formation and liver metastasis

**DOI:** 10.1038/s41421-025-00819-8

**Published:** 2025-08-12

**Authors:** Pu Tian, Qiuyao Wu, Dasa He, Wenjing Zhao, Lichao Luo, Zhenchang Jia, Wenqian Luo, Xianzhe Lv, Yanan Liu, Yuan Wang, Qian Wang, Peiyuan Zhang, Yajun Liang, Qifeng Yang, Guohong Hu

**Affiliations:** 1 CAS Key Laboratory of Tissue Microenvironment and Tumor, Shanghai Institute of Nutrition and Health, University of Chinese Academy of Sciences, Chinese Academy of Sciences, Shanghai, China; 2 Department of Breast Surgery, Qilu Hospital of Shandong University, Ji’nan, Shangdong China

**Keywords:** Cancer microenvironment, Metastasis

## Abstract

The liver is a major target organ for breast cancer metastasis, while the regulatory mechanism of liver colonization by breast cancer remains largely unclear. Neutrophils are known to play important roles in metastatic colonization of cancer cells by the formation of neutrophil extracellular traps (NETs). Here we show the role and mechanism of a subpopulation of Kupffer cells (KCs), the liver resident macrophages, in mediating tumoral induction of NETs and liver metastasis. NETs are activated more abundantly in liver metastases of breast cancer, as compared to metastases to other organs and primary tumors. Liver-tropic tumor cells induce CD62L-expressing KCs by a secretory protein DMBT1, and CD62L^+^ KCs activate neutrophils for NETosis via the chemokine CCL8. Inhibition of CCL8 or its receptor on neutrophils, CCR1, impairs NETosis and metastasis. In addition, we identified a KC membrane protein MUC1 that binds to DMBT1 and subsequently activates NF-κB signaling in KCs, leading to CCL8 and CD62L expression. KCs with MUC1 inhibition effectively suppress liver metastasis. Furthermore, a DMBT1 neutralizing antibody was developed with the promise to inhibit tumor–KC interaction and treat metastatic cancer. In conclusion, our work reveals a KC subset that accounts for the liver tropism of breast cancer cells and NETs, and provides potential strategies in metastasis treatment.

## Introduction

The majority of cancer-related mortality is attributed to metastasis. The liver is one of the most frequent target organs of breast cancer metastasis, and hepatic relapse is often associated with a very poor prognosis^1,2^. In clinical practice, the patients are usually not suitable for surgery when liver metastasis is diagnosed, and options of systemic treatment are also limited. The mechanisms underlying liver tropism of breast cancer remain poorly understood, hindering the rational design of systemic therapeutics. Metastatic colonization of tumor cells is often supported by microenvironmental components in target organs^3,4^. The liver-resident components mainly include hepatocytes, liver sinusoidal endothelial cells, stellate cells and Kupffer cells (KCs). In addition, many types of immune cells, such as macrophages, neutrophils and lymphocytes, are present during tumor dissemination and colonization in the liver^5^. Identifying the crucial populations of these cells that affect liver metastasis and understanding how they communicate with disseminated tumor cells can be instrumental for therapeutic development.

Neutrophils are the most abundant leukocytes in the circulation and the first line of defense in response to infection or injury^6^. They play their roles in host defense by phagocytosis, degranulation and formation of neutrophil extracellular traps (NETs), the web-like extracellular structures composed of decondensed chromatin and cytotoxic granule-derived proteins that restrain and kill invading microbes^7,8^. Recently, it has been found that NET formation, i.e., NETosis, can also be induced by tumors and NETs in turn support tumor progression and metastasis^9–18^. NETs often play tumor-fostering roles in cancer growth and spreading, stromal and vascular remodeling, immune suppression and metabolic rewiring^13–18^. Such effects of NETs are mediated by the NET-bound proteins, such as myeloperoxidase (MPO), neutrophil elastase (NE), cathepsin G (CTSG) and matrix metalloproteinase 9 (MMP9), or the NET DNA, which is enriched with 8-hydroxy-2’-deoxyguanosine. NETs are observed in multiple tumor-bearing organs, such as the lungs, liver and kidney^10^, and interestingly, are particularly abundant in liver tumors and metastases^9,18,19^. NET targeting effectively suppresses liver metastasis of breast cancer and colorectal cancer^18,20^. However, it is less clear how tumor induces NETosis in these organs.

KCs are a group of tissue-resident macrophages abundantly localized in the sinusoids of the liver with essential roles to scavenge microorganisms, clear metabolic waste, induce immunological tolerance and respond to tissue damage^21,22^. In addition to their contribution to the maintenance of hepatic and systemic homeostasis, these professional phagocytes are also involved in tumor growth and metastasis in the liver, with studies showing both anti- and pro-tumor effects of KCs in colorectal cancer, pancreatic cancer and hepatocarcinoma^22–27^. KCs participate in tumoral exosomes-induced pre-metastatic niches in the liver for pancreatic cancer metastasis^25^, but can also suppress tumor growth and metastasis by phagocytizing cancer cells and orchestrating anti-tumor immunity^27,28^. Notably, in vivo engineering of liver macrophages by lentiviral delivery of IFNα^29^ or bacterial delivery of CRISPR/Cas9 gene-editing machinery^26^ reprograms KCs and effectively inhibits liver metastasis of various cancers. These studies demonstrate the heterogeneity of KC populations in tumor microenvironment and indicate the promise of KC targeting for cancer treatment. However, the role and regulation of KC heterogeneity in breast cancer metastasis are yet to be investigated.

## Results

### NETs are abundantly present in the liver and promote breast cancer metastasis

**Fig. 1:**
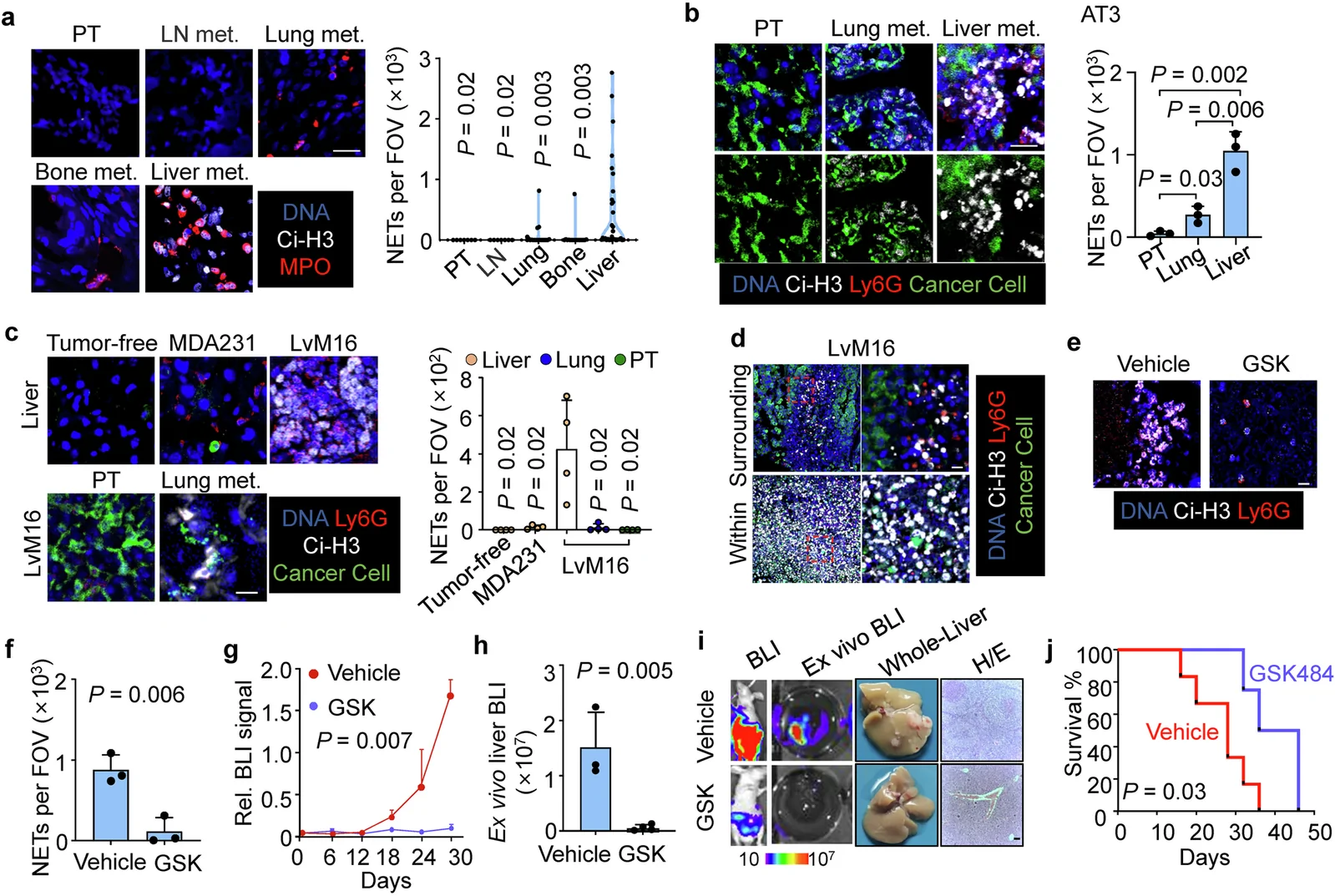
NETs are enriched in the liver and promote metastases of breast cancer. **a** NET abundance by IF analysis of Ci-H3 and MPO in primary tumor (PT) and LN, lung, bone and liver metastases (met.) of human breast cancer in the Qilu cohort. *P* values are calculated in comparison to liver metastases. **b** NET abundance in primary tumors, lung and liver metastases of C57BL/6 mice at week 6 after orthotopic injection of GFP-labeled AT3 cells. **c** NET abundance in livers of nude mice with intrasplenic injection of MDA-MB-231 (MDA231) and its liver-tropic subline LvM16 on day 16 after inoculation (top), or in primary tumors with LvM16 orthotopic inoculation on day 50 and in lung with intravenous LvM16 inoculation (bottom) on day 30. *P* values are calculated in comparison to LvM16 liver metastases. **d** NETs surrounding or within metastatic foci in livers of nude mice on day 40 after intrasplenic injection of LvM16 (zoomed areas shown on right). **e**–**j** GSK484 (GSK) treatment (20 mg/kg) of nude mice after LvM16 intrasplenic inoculation for liver metastasis analyses. Shown are IF analysis of NETs in livers (**e**, **f**), in vivo bioluminescent imaging (BLI) signals of livers (**g**, signal normalized to day 0), ex vivo BLI signals of livers (**h**), representative images of BLI and liver metastases (**i**), and overall survival of the mice (**j**). *n* = 7 (PT/LN), 20 (lung), 17 (bone) or 33 (liver) patients (**a**), or 3 (**b**, **f**, **h**), 4 (**c**), or 6 mice (**g**, **j**) per group. *P* values were obtained by repeated measures two-way ANOVA (**g**), Log rank test (**j**) and two-tailed unpaired *t*-test (others). Scale bars, 20 μm. Data are shown as mean ± SEM (**g**) or mean ± SD (others).

The pro-tumor roles of NETs have been well established. To investigate the presence of tumor-associated NETs in different organs, breast cancer clinical samples from a Qilu cohort, including primary tumors, metastases of lymph nodes (LNs), lungs, bone and liver (Supplementary Fig. S1a) were analyzed by immunofluorescence (IF) staining with NET biomarkers MPO and citrullinated histone H3 (Ci-H3). NETs were scarcely detected in primary tumors or LNs, but readily observed in distant metastases of bone, lungs and liver. Particularly, NETs were most abundant in liver metastases (Fig. 1a), corroborating the previous finding by Yang et al.^18^.

Thus, we further analyzed NETs in breast cancer in mice. The murine breast cancer cells AT3 were inoculated into mice orthotopically, and metastases were developed in both lungs and liver after 6 weeks. NETs were observed in liver and lung metastases, but not in primary tumors. Importantly, much more abundant NETs were present in the liver than in lungs (Fig. 1b). The same pattern of NETosis in different organs was also observed in mice bearing the Py8119 breast tumors, as abundant NETs appeared in the liver after intrasplenic inoculation, but fewer were observed in primary tumors and lung metastases after orthotopic or intravenous inoculation (Supplementary Fig. S1b). We further analyzed NETs in metastases caused by human breast cancer cells. Due to the lack of liver-metastatic human breast cancer cell lines, we first performed a repeated in vivo selection of MDA-MB-231 cells for liver-tropic sublines with mammary fat-pad and intrasplenic injection in nude mice (Supplementary Fig. S1c). The selection resulted in a moderately (LvM4) and a strongly (LvM16) liver-metastatic subline of MDA-MB-231 (Supplementary Fig. S1d, e). When LvM16 cells were transplanted intrasplenically into the mice, NETs were observed around tumor cells in the liver on day 16 after transplantation (Fig. 1c). Abundant NETs were present surrounding or within tumor foci when metastases were established on day 40 (Fig. 1d). By contrast, NETs were rarely seen in livers of tumor-free mice or those bearing MDA-MB-231 cells (Fig. 1c), which could not colonize the liver for metastasis (Supplementary Fig. S1d, e). In addition, the NET-inducing capacity of LvM16 was also weaker in the mammary fat pad or lung when cells were orthotopically or intravenously inoculated into mice (Fig. 1c).

To confirm the role of neutrophils and NETs in liver metastasis, we first used an anti-Ly6G antibody^30^ to deplete neutrophils in mice (Supplementary Fig. S1f) and found that neutrophil depletion led to suppression of liver metastasis by LvM16 cells (Supplementary Fig. S1g, h). In another assay, the LvM16-bearing mice were treated with the histone citrullination inhibitor GSK484 or DNase I to suppress NETs in the liver (Fig. 1e, f; Supplementary Fig. S1i). NET inhibition also effectively impeded liver metastasis (Fig. 1g–i; Supplementary Fig. S1j, k) and extended animal survival (Fig. 1j). These data corroborate the enrichment of pro-tumor NETs in liver metastases of breast cancer.

### A CD62L^+^ KC subset in liver metastasis

We hypothesized that the enrichment of NETs in the liver is attributed to some local components of the liver microenvironment. To find out the early changes in the liver that cause NETosis, a temporal analysis of NETosis was performed after LvM16 inoculation, and it was observed that NETs appeared in the liver on day 7 (Supplementary Fig. S2a). Thus, the livers were analyzed by single-cell transcriptomic sequencing (scRNA-seq) on day 7 after intrasplenic inoculation of MDA-MB-231 or LvM16. As NETosis is an immune response, we focused on the CD45^+^ immune cells in the scRNA-seq analysis. Totally 12,025 cells (5225 and 6800 cells for MDA-MB-231 and LvM16 samples, respectively) were analyzed, and 6 major cell types including B cells, monocytes/macrophages, neutrophils, mast cells, natural killer (NK) cells and dendritic cells (DCs), were identified (Supplementary Fig. S2b, c). Notably, only a portion of the designated neutrophils expressed *LY6G* (Supplementary Fig. S2d), consistent with previous studies indicating the technical difficulties in analyzing these short-lived cells by regular scRNA-seq approaches^31,32^. Thus, we focused on other cell types that could participate in neutrophil regulation for NETosis.

**Fig. 2:**
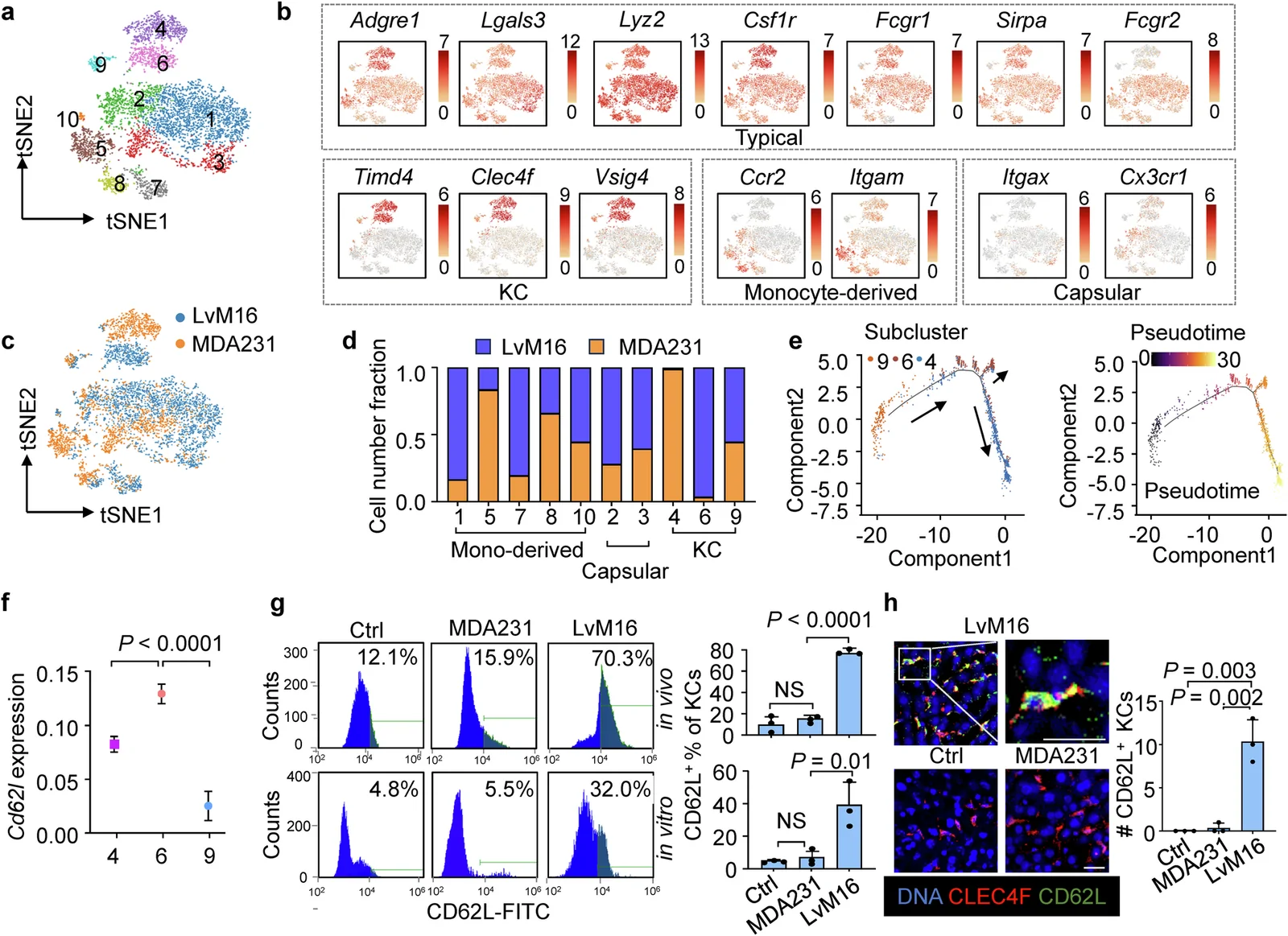
CD62L^+^ Kupffer cells are enriched in early liver metastatic niche. **a**–**f** scRNA-seq analyses of macrophages in livers of nude mice on day 7 after intrasplenic injection of MDA-MB-231 or LvM16. Shown are *t*-SNE map of 10 macrophage subclusters (**a**), expression of typical marker genes of various macrophage types in the subclusters (**b**), *t*-SNE distribution (**c**) and abundance (**d**) of macrophage subclusters in MDA-MB-231 and LvM16-inoculated livers, unsupervised transcriptional trajectory of KC differentiation, colored by cell clusters or pseudotime (**e**), and *Cd62l* expression levels in KC subclusters 4, 6 and 9 (**f**). **g** Flow cytometry analyses of CD62L^+^ KC abundance in livers of mice inoculated with PBS or different cancer cells (top, *n* = 3 mice), and in primary KCs treated by CM of different cancer cells (bottom, *n* = 3 biological repeats). **h** IF analysis of CD62L^+^ KCs in livers inoculated with PBS or different cancer cells (zoomed areas shown at bottom). *P* values were obtained by two-tailed unpaired *t*-test; NS, not significant. Scale bars, 50 μm. Data are shown as mean ± SEM (**f**) or mean ± SD (others).

Comparing MDA-MB-231 and LvM16-inoculated livers revealed no significant changes in total abundance of the major immune cell types (Supplementary Fig. S2c). Then we analyzed the changes in heterogeneity of these cells. Except for the mast cells which were in low cell numbers, other types of cells could be further allocated into subclusters by *t*-SNE analysis. Although slight differences in the relative abundance of DC, B and NK cell subclusters were observed, there were no obvious changes in the overall distribution of these cell subclusters in MDA-MB-231 and LvM16-inoculated livers (Supplementary Fig. S2e–g). However, evident differences in monocyte/macrophage distribution were found. The monocytes/macrophages could be split into 10 subclusters (Fig. 2a; Supplementary Fig. S2h), with all of them expressing the typical macrophage markers, including *ADGRE1 (*encoding F4/80*), LGALS3, LYZ2, CSF1R, FCGR1, SIRPA* and *FCGR2*. Subclusters 1, 5, 7, 8 and 10 additionally expressed high levels of *CCR2* and *ITGAM* and thus were designated as monocyte-derived macrophages. Subclusters 2 and 3 expressed the markers of capsular macrophages *ITGAM* and *CX3CR1*^33^. Subclusters 4, 6, 9 expressed *TIMD4, CLEC4F* and *VSIG4* and thus were determined as KCs (Fig. 2b). The distribution of several macrophage subclusters in the liver was significantly altered, with the most obvious changes observed in KCs. KC subcluster 4 was exclusively present in the liver with MDA-MB-231, but subcluster 6 appeared predominantly in the LvM16-inoculated liver (Fig. 2c, d). KC subcluster 9, with a transcriptional profile closer to that of normal KCs from tumor-free mice (Supplementary Fig. S2i), was evenly distributed in both conditions (Fig. 2d). A pseudotime analysis showed a differentiation path from subcluster 9 to subcluster 4 or 6 (Fig. 2e), indicating an influence of liver-tropic cancer cells on KC polarization into subcluster 6. Comparison of the expression of cell surface protein-encoding genes in the KC subclusters revealed that *CD62L*, encoding the transmembrane glycoprotein L-selection which is usually expressed in lymphoid-primed hematopoietic stem cells and circulating leukocytes^34–36^, ranked at the top of upregulated genes in subcluster 6 (Fig. 2f; Supplementary Fig. S2j and Table S1). We analyzed the abundance of KC subcluster 6, with CD62L as a marker, in murine livers by flow cytometry and IF analyses, and found that CD62L^+^ KCs were obviously enriched in LvM16-inoculated livers compared to tumor-free tissues or those with non-metastatic cancer cells (Fig. 2g, h), consistent with the scRNA-seq analysis. Importantly, culturing primary KCs isolated from healthy mice in conditioned medium (CM) of cancer cells showed that LvM16 could induce KC polarization into the CD62L^+^ population (Fig. 2g). Temporal analyses of KCs in murine livers showed the enrichment of CD62L^+^ cells beginning on day 7 after LvM16 inoculation (Supplementary Fig. S2k), a time point coincident with the appearance of NETs (Supplementary Fig. S2a). Thus, we speculated that CD62L^+^ KCs might play a role in NET formation and metastasis in the liver.

### CD62L^+^ KCs promote liver metastasis of breast cancer cells

**Fig. 3:**
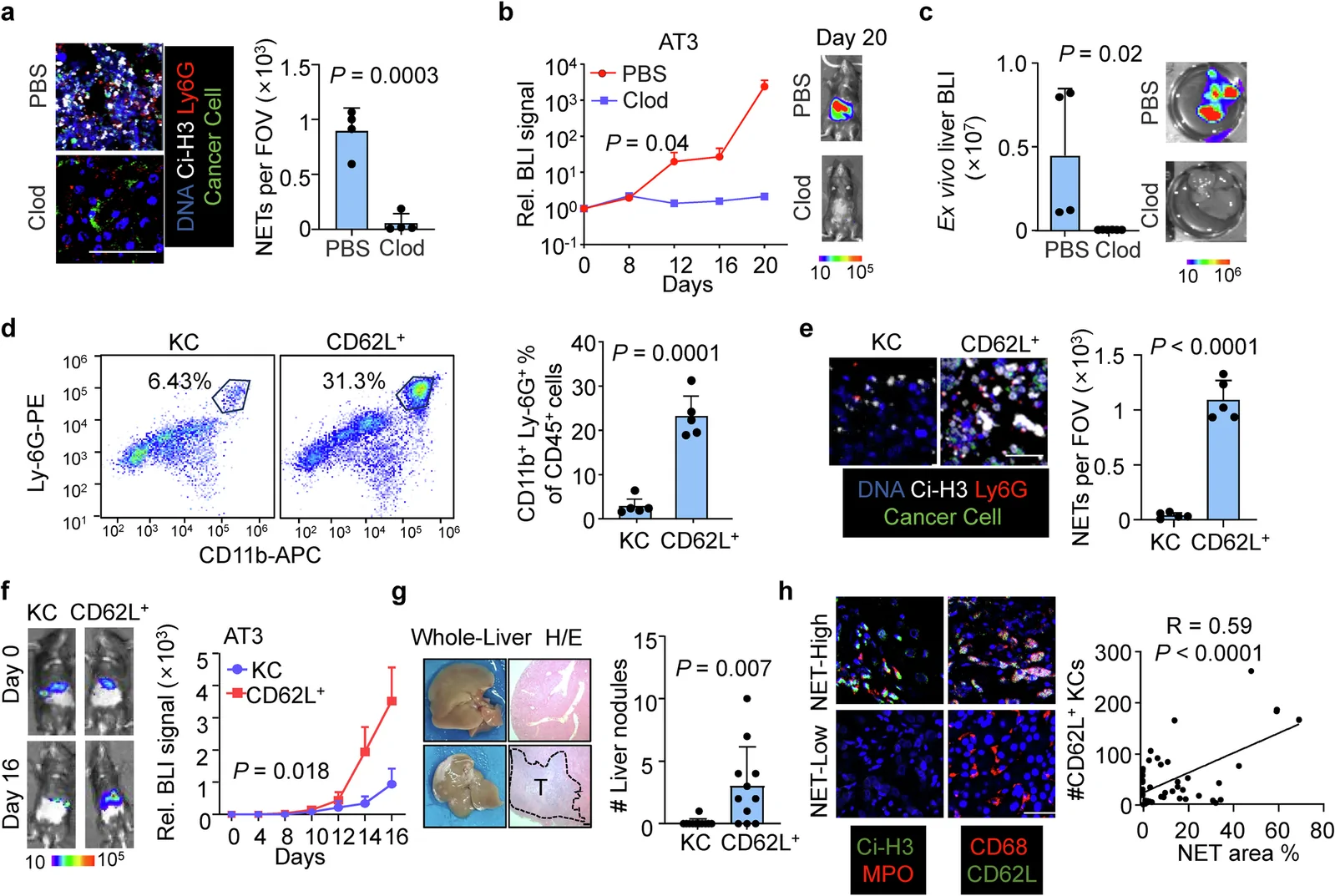
CD62L^+^ KCs promote liver metastasis of breast cancer. **a**–**c** The effect of Clod treatment of C57BL/6 mice with intrasplenic inoculation of AT3 cells. Shown are IF analyses of NETs in livers (**a**), in vivo (**b**) and ex vivo (**c**) BLI of livers. **d**–**g** Intrasplenic injection of 1:5 mixture of AT3 with total KCs or CD62L^+^ KCs in C57BL/6 mice for metastasis analysis. Shown are flow cytometry analyses of CD11b^+^Ly6G^+^ neutrophils (**d**) and NETosis (**e**) in livers, in vivo BLI (**f**) and liver metastatic nodules (**g**). **h** IF analyses of NETosis and CD62L^+^ KC correlation in liver metastases of Qilu human breast cancer sample. NETs and CD62L^+^ KCs are stained separately. *n* = 4 (**a**–**c**), 5 (**d,** **e**) or 11(**f**, **g**) mice per group and 23 patients (**h**). *P* values were obtained by repeated measures two-way ANOVA (**b**, **f**), Pearson correlation analysis (**h**) or two-tailed unpaired *t*-test (others). Scale bars, 50 μm. Data are shown as mean ± SEM (**b**, **f**) or mean ± SD (others).

To investigate whether KCs are involved in NETosis and liver metastasis, the mice were treated with clodronate liposomes (Clod) to deplete KCs^37,38^, followed by cancer cell inoculation. The treatment effectively cleared KCs in the liver (Supplementary Fig. S3a, b). Importantly, KC depletion significantly suppressed AT3-induced NETosis (Fig. 3a), and dampened the metastatic growth of cancer cells (Fig. 3b, c; Supplementary Fig. S3c). Notably, Clod treatment led to no obvious changes in infiltration of CD8^+^ or CD4^+^ T cells (Fig. S3d), suggesting that KC clearance had no direct effect on T cell-mediated anti-tumor immunity.

Then we further assessed the role of CD62L^+^ KCs. Primary KCs were isolated by Percoll gradient centrifugation (Supplementary Fig. S3e), followed by culturing in LvM16 CM and flow cytometry purification of the CD62L^+^ subpopulation. Further primary KCs or CD62^+^ KCs were mixed with AT3 cancer cells in a ratio of 5:1 and intrasplenically transplanted into mice (Supplementary Fig. S3f). Compared to the unspecialized KCs, CD62L^+^ KCs led to enhanced neutrophil infiltration in the liver (Fig. 3d) and promoted NETosis (Fig. 3e). More importantly, co-injection of CD62L^+^ KCs with cancer cells accelerated cancer colonization in the liver, resulting in more metastatic nodules (Fig. 3f, g; Supplementary Fig. S3g). Furthermore, IF staining of liver metastases of human breast cancer demonstrated a positive correlation of CD62L^+^ KC abundance and NETosis (Fig. 3h). These data confirmed the involvement of CD62L^+^ KCs in NETosis and liver metastasis.

### CD62L^+^ KCs induce NETosis by CCL8

**Fig. 4:**
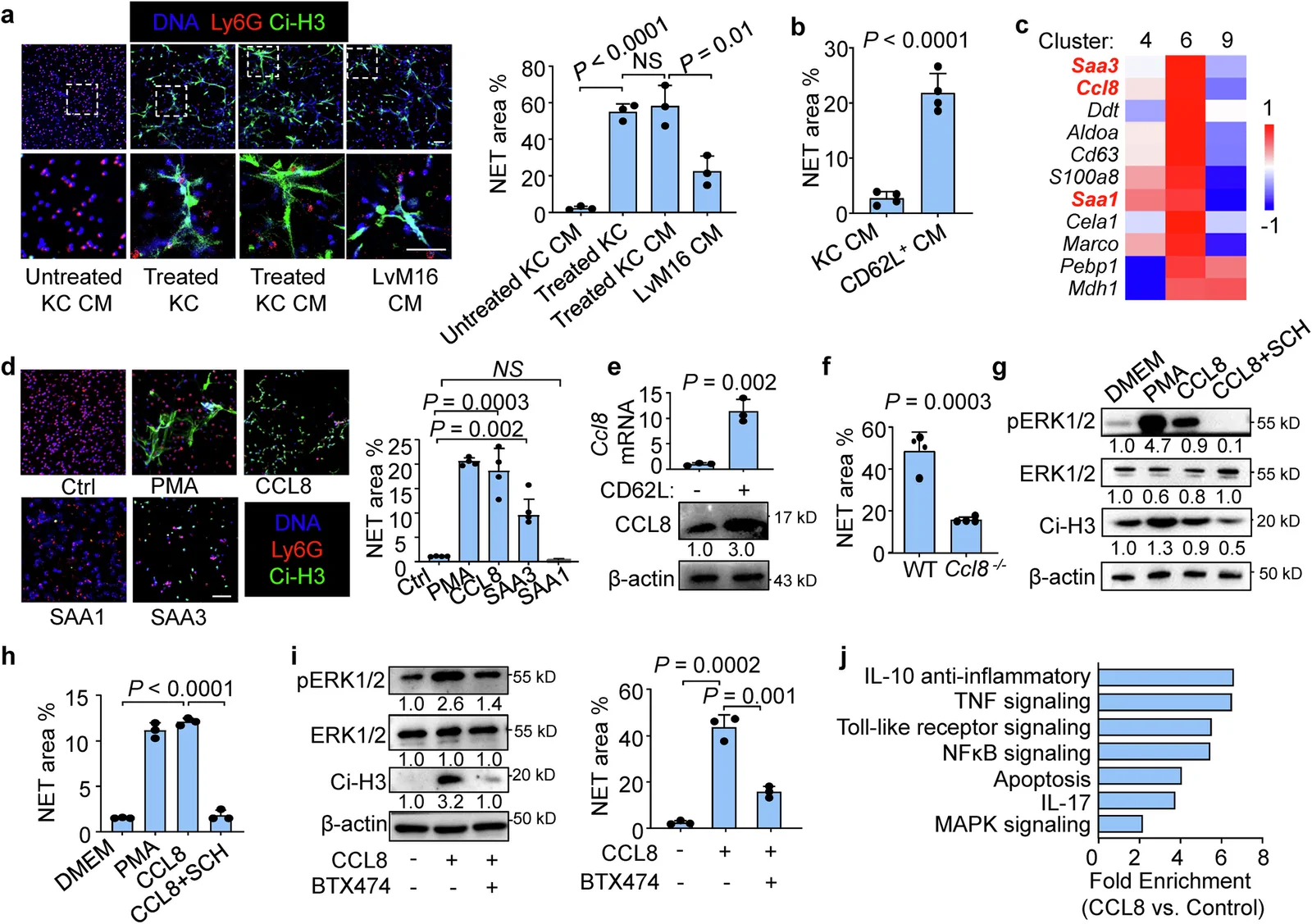
CD62L^+^ KCs induce NETosis by CCL8. **a** NETosis of murine primary neutrophils cultured with CM of untreated KCs, KCs pretreated with LvM16 CM, or CM of the pretreated KCs, or CM of LvM16 (zoomed areas shown at bottom). **b** NETosis of murine primary neutrophils cultured in CM of total KCs or CD62L^+^ KCs. **c** Expression heatmap of secreted protein-encoding genes in KC subclusters. **d** NETosis of neutrophils cultured with recombinant CCL8 (1 ng/mL), SAA1 or SAA3 (10 ng/mL) or PMA (20 nM) for 16 h. **e** mRNA and protein expression of *Ccl8* in CD62L^+^ and CD62L^–^ KCs isolated from LvM16 liver metastases of mice. Relative quantitation of blot intensity normalized to loading control was provided below each blot. **f** NETosis of neutrophils cultured with CM of KCs from WT or *Ccl8*^–/–^ mice. The KCs were pretreated with CM of LvM16. **g**, **h** ERK phosphorylation and NETosis quantitation by IF analysis of murine neutrophils treated with PMA, recombinant CCL8 (10 ng/mL) and/or the ERK inhibitor SCH772984 (SCH, 1 μM) for 16 h. **i** ERK phosphorylation and NETosis of neutrophils treated with CCL8 (10 ng/mL) and/or the CCR1 antagonist BX471 (1 nM) for 16 h. **j** GO analyses of the upregulated genes in rCCL8-treated vs untreated neutrophils. *n* = 3 (**a**, **e**, **h**, **i**), 4 (**b**, **d**, **f**) biological repeats per group. Scale bars, 50 μm. *P* values were obtained by two-tailed unpaired *t*-test. Data are shown as mean ± SD.

Co-culturing murine neutrophils isolated from bone marrow (Supplementary Fig. S4a) with KCs pre-treated with LvM16 CM, but not untreated KCs, led to obvious NETosis (Fig. 4a). Notably, the CM of LvM16-pretreated KCs exerted a similar effect, while directly treating neutrophils with LvM16 CM was much less effective in inducing NETosis (Fig. 4a). The CM of purified CD62L^+^ KCs was also more potent than that of unspecified KCs for NET induction (Fig. 4b). These data indicated that tumor-induced CD62L^+^ KCs promote NETosis via some secretory factors. Thus, we analyzed the scRNA-seq data of KCs, and found a list of upregulated genes encoding secretory factors in KC subcluster 6 vs other KCs (Fig. 4c). Among these genes, *Ccl8*, *Saa3* and *Saa1* were attractive candidates as these are all pro-inflammatory factors^39–41^ and NETosis is largely an inflammatory response of neutrophils. However, their roles in NETosis are unknown. Therefore, we tested whether the recombinant proteins of CCL8, SAA1 or SAA3 could activate neutrophils for NETosis. It turned out that CCL8 and SAA3, but not SAA1, induced NETosis of murine primary neutrophils. In addition, CCL8 demonstrated a dose-dependent and much more potent effect on activating NETosis than SAA3, with the efficacy comparable to phorbol myristate acetate (PMA), a known NET inducer (Fig. 4d; Supplementary Fig. S4b). CCL8 was only able to induce neutrophils for NETosis, but could not recruit neutrophils (Supplementary Fig. S4c). We further observed higher *Ccl8* expression in KCs isolated from LvM16 liver metastases as compared to macrophages in liver metastases and in tumor-free livers (Supplementary Fig. S4d), and in CD62L^+^ KCs as compared to CD62L^–^ KCs (Fig. 4e). In human liver metastases of breast cancer, CCL8 expression was also associated with the presence of NETs (Supplementary Fig. S4e). More importantly, KCs derived from *Ccl8*-knockout mice, even pretreated with LvM16 cancer cells, demonstrated a much weaker capacity to activate neutrophils than the wild-type (WT) counterpart (Fig. 4f).

CCL8 is a chemokine that binds to the CCR family receptors, including CCR1, CCR2, CCR3 and CCR5, and activates the downstream ERK signaling pathway, which is known to be crucial for NETosis of neutrophils^42–44^. Among these CCL8 receptors, *Ccr1* is highly expressed in neutrophils (Supplementary Fig. S4f). CCL8 treatment activated ERK signaling in neutrophils, while the CCR1 antagonist BX471 and the ERK inhibitor SCH772984 both suppressed ERK activation, leading to ablation of CCL8-induced histone citrullination and NET formation of neutrophils (Fig. 4g–i). In particular, SCH772984 demonstrated a dose-dependent effect on NETosis inhibition (Supplementary Fig. S4g, h). *Ccr1* knockdown by siRNA in neutrophils also inhibited CCL8-induced ERK activation and histone citrullination (Supplementary Fig. S4i). Interestingly, *Ccr1* expression in neutrophils could be upregulated by CCL8 treatment and suppressed by ERK inhibition (Supplementary Fig. S4j), suggesting a positive feedback on *Ccr1* expression and the downstream ERK signaling. We further investigated CCL8-induced molecular changes in neutrophils by transcriptomic sequencing and observed the upregulation of TNF, NF-κB and apoptosis signaling pathways (Fig. 4j), which was similar to that resulting from PMA treatment (Supplementary Fig. S4k) and concordant with the changes during NETosis^10,45^. The changes of genes in these signaling pathways were further validated by quantitative PCR analysis and importantly, ERK inhibition reversed these CCL8-induced changes (Supplementary Fig. S4l), corroborating that CCL8 induces NET formation of neutrophils by ERK signaling activation.

### Tumor-derived DMBT1 induces CD62L^+^ KCs and promotes liver metastasis

**Fig. 5:**
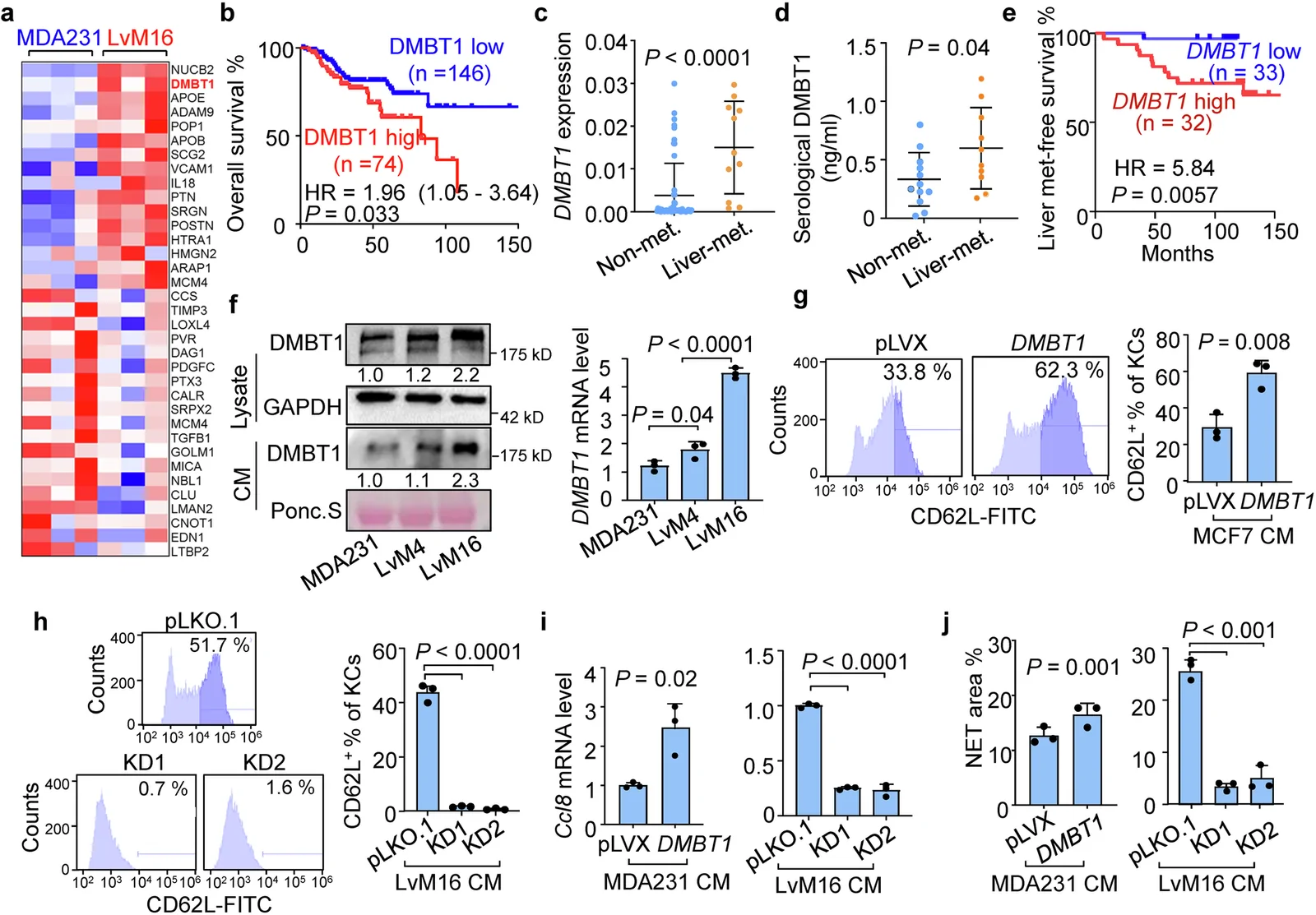
Tumor induces CD62L^+^ KCs by DMBT1. **a** Heatmap of secreted protein-encoding genes differentially expressed in MDA-MB-231 vs LvM16. **b** Overall survival of breast cancer patients stratified by *DMBT1* expression in the UNC cohort (GSE3521, GSE26338). **c**–**e** DMBT1 expression in human breast cancer of the Qilu cohort. Shown are tumoral *DMBT1* mRNA levels (**c**) and serological DMBT1 protein levels (**d**) of those with liver-metastatic or non-metastatic primary tumors, and liver metastasis-free survival of patients stratified by tumoral *DMBT1* expression (**e**). **f** *DMBT1* mRNA and protein expression in MDA-MB-231 sublines. **g**–**i** CD62L induction (**g**, **h**) and *Ccl8* expression (**i**) of KCs treated with CM of the indicated tumor cells with or without *DMBT1* overexpression or knockdown. **j** NETosis of neutrophils cultured with CM from the KCs in **i**. *n* = 220 (**b**), 65 (**c**, **e**) and 22 (**d**) patients, and 3 biological repeats (**f**–**j**). *P* values were obtained by log-rank test (**b**, **e**) or two-tailed unpaired *t*-test (others). Data are shown as mean ± SD.

As the CM of liver-tropic cancer cells could induce CD62L^+^ KCs (Fig. 2g), we attempted to investigate what tumor-derived extracellular factors induce CD62L^+^ KCs. When the CM contents of MDA-MB-231 and LvM16 were separated by ultrafiltration centrifugation into two fractions of different molecular sizes, it was observed that the > 3 kilo Dalton (kDa) fraction of LvM16 CM demonstrated an effect for KC polarization similar to that of the total CM. By contrast, the < 3 kDa fraction of LvM16 or both fractions of MDA-MB-231 CM were unable to induce CD62L^+^ KCs (Supplementary Fig. S5a). These results indicated that the secreted proteins, instead of small molecules like metabolites, played a major role in KC polarization. Thus, we analyzed the secreted proteins of MDA-MB-231 and LvM16 by mass spectrometric profiling. Among the upregulated secreted proteins in LvM16, nucleobindin 2 (NUCB2) and human deleted in malignant brain tumors 1 (DMBT1) ranked at the top (Fig. 5a). Further analysis of their expression in human breast cancer samples of a published UNC dataset^46,47^ showed the association of *DMBT1*, but not *NUCB2*, expression with patient survival (Fig. 5b; Supplementary Fig. S5b). In addition, analysis of an in-house Qilu breast cancer cohort revealed higher *DMBT1* expression in liver-metastatic breast tumors than non-metastatic tumors (Fig. 5c). Serological DMBT1 protein levels were also higher in the patients with tumors prone to liver metastasis (Fig. 5d). Higher *DMBT1* expression in primary tumors was associated with accelerated liver metastasis in these patients (Fig. 5e). These data suggest a role of DMBT1 in liver metastasis.

DMBT1 is a member of scavenger receptor superfamily and is involved in immunological response to infection^48–51^, but its role in metastasis and KC regulation is unknown. We first validated its upregulation of expression and secretion in liver-tropic sublines of MDA-MB-231 (Fig. 5f). In murine breast cancer cell lines, *Dmbt1* expression was also higher in the liver-tropic AT3 and Py8119 as compared to the less metastatic NF639 and 4TO7 cells (Supplementary Fig. S5c, d). Meanwhile, the CM of AT3 and Py8119 was more potent in inducing CD62L^+^ differentiation of primary KCs than that of NF639 and 4TO7 (Supplementary Fig. S5e). In addition, *DMBT1* overexpression in both MDA-MB-231 and MCF7 (Supplementary Fig. S5f, g) promoted the capacity of tumoral CM to induce CD62L^+^ polarization and *Ccl8* expression of KCs (Fig. 5g–i; Supplementary Fig. S5h). Importantly, the KCs treated by DMBT1-containing CM demonstrated enhanced capacity to induce neutrophils for NET formation (Fig. 5j). Reciprocally, *DMBT1* knockdown in LvM16 (Supplementary Fig. S5f, g) impaired the capacity of LvM16 CM to induce CD62L^+^ polarization and *Ccl8* expression of primary KCs (Fig. 5h, i), leading to suppressed NET formation in vitro when neutrophils were co-cultured with tumor-pretreated KCs (Fig. 5j). Meanwhile, *DMBT1* knockdown in murine AT3 (Supplementary Fig. S5i) also inhibited tumor-induced CD62L^+^ polarization (Supplementary Fig. S5j).

**Fig. 6:**
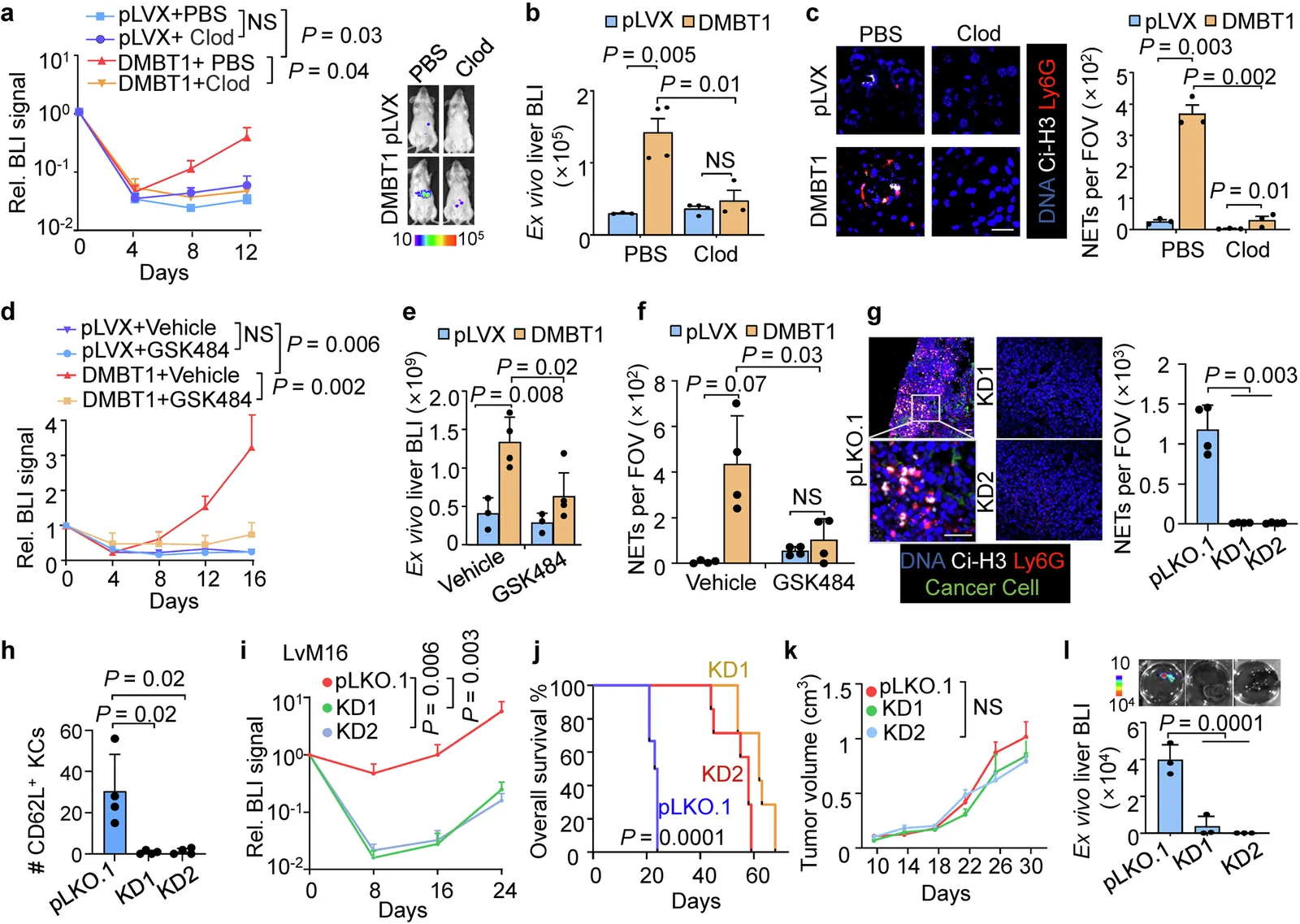
DMBT1 promotes liver metastasis of breast cancer. **a**–**c** In vivo BLI (**a**), ex vivo BLI (**b**) and NETosis (**c**) in livers of NOD-SCID mice treated with Clod after intrasplenic inoculation of MCF7 with or without *DMBT1* overexpression. **d–f** In vivo BLI (**d**), ex vivo BLI (**e**) and NETosis (**f**) of NOD-SCID mice treated with GSK484 after intrasplenic inoculation of MCF7 with or without *DMBT1* overexpression. **g**–**j** NETosis (**g**), CD62L^+^ KCs (**h**) and in vivo BLI (**i**) of livers, and survival (**j**) of nude mice after intrasplenic injection of LvM16 cells with *DMBT1* knockdown. **k**, **l** Primary tumor growth (**k**) and liver metastasis (**l**) in C57BL/6 mice after orthotopic injection of AT3 cells with *Dmbt1* knockdown. *n* = 3 or 4 (**a**, **b**), 3 (**c**, **h**, **k**, **l**), 7 (**d**, **i**, **j**), 4 (**e**, **f**, **g**) mice per group. *P* values were obtained by repeated measures two-way ANOVA (**a**, **d**, **i**, **k**), Log rank test (**j**) or two-tailed unpaired *t*-test (others). Scale bars, 50 μm. Data are shown as mean ± SEM (**a,** **d,** **i,** **k**) or mean ± SD (others).

The effect of tumor-derived DMBT1 on cancer metastasis was then assessed *DMBT1* overexpression and knockdown did not cause obvious changes in cancer cell growth, invasion or apoptosis (Supplementary Fig. S6a–d). However, when MCF7 cells with *DMBT1* overexpression were inoculated into NOD-SCID mice, metastatic growth in the liver was significantly enhanced (Fig. 6a, b; Supplementary Fig. S6e). This was accompanied by increased neutrophil infiltration (Supplementary Fig. S6f) and NET formation (Fig. 6c). The effect of *DMBT1* was dependent on KCs, as Clod treatment to deplete KCs in the liver (Supplementary Fig. S6g) effectively suppressed NET formation and liver metastasis of *DMBT1*-overexpressing cancer cells (Fig. 6a–c; Supplementary Fig. S6e).

Further, treating the mice with GSK484 suppressed NETosis and blocked *DMBT1*-induced liver metastasis (Fig. 6d–f). By contrast, *DMBT1* knockdown in LvM16 cells significantly suppressed CD62L^+^ KC induction, neutrophil infiltration and NETosis in the liver after intrasplenic transplantation (Fig. 6g, h; Supplementary Fig. S6h, i), resulting in alleviated metastatic burden (Fig. 6i; Supplementary Fig. S6j, k) and extended survival of the mice (Fig. 6j). In addition, *Dmbt1* knockdown in Py8119 cells (Supplementary Fig. S6l) also led to suppression of NETosis and metastasis in immunocompetent C57BL/6 mice after intrasplenic inoculation (Supplementary Fig. S6m, n). Importantly, treating the mice with the DMBT1-containing Py8119 CM could partially rescue the effect of *Dmbt1* knockdown, corroborating the role of secreted DMBT1 in regulating liver metastasis. Similarly, *Dmbt1* knockdown in AT3 also led to inhibition of CD62L^+^ KC polarization and liver metastasis after intrasplenic inoculation (Supplementary Fig. S6o, p), but did not affect T cell infiltration in metastases (Supplementary Fig. S6q). We also analyzed the role of DMBT1 in spontaneous liver metastasis. When AT3 cells were orthotopically xenografted, *Dmbt1* knockdown had no effects on primary tumor growth (Fig. 6k), but suppressed NET formation and metastatic growth in the liver (Fig. 6l; Supplementary Fig. S6r). In addition, multiplex IF staining of human liver metastases of breast cancer confirmed the correlation of NETs with CD68^+^ cells, DMBT1 and CCL8 expression (Supplementary Fig. S6s, t).

### DMBT1 regulates KCs by the MUC1-NF-κB signaling pathway

**Fig. 7:**
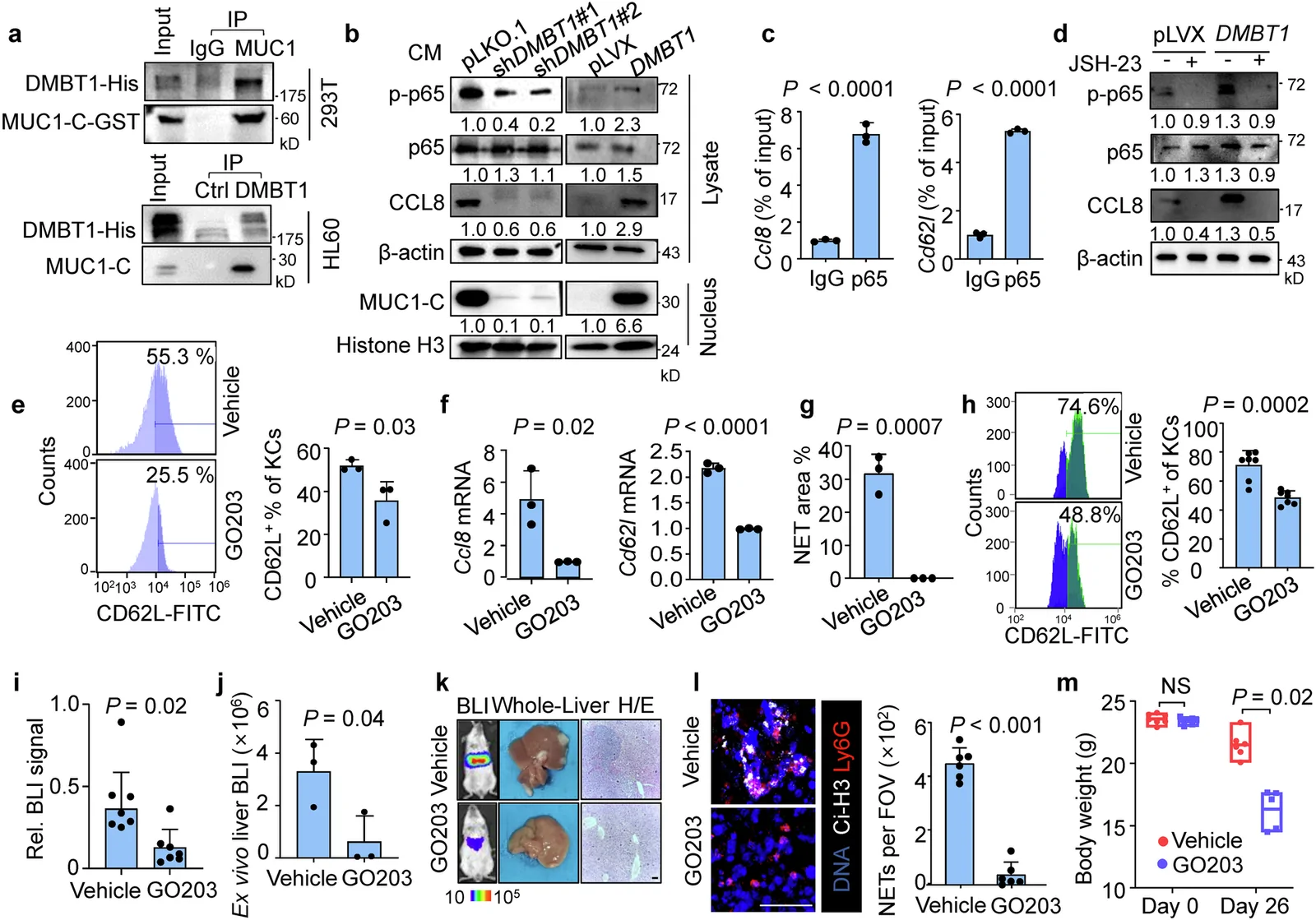
DMBT1 regulates KCs via MUC1-NF-κB signaling. **a** Co-IP assays for DMBT1 and MUC1-C in 293 T and HL60 cells. Ctrl, Ni-NTA bead control sample. **b** p65 phosphorylation and nuclear distribution of MUC1-C in KCs after treatment of CM of cancer cells with or without *DMBT1* knockdown/overexpression. **c** ChIP-qPCR analysis of p65 binding to *Ccl8* or *Cd62l* promoters in monocytes. **d** p65 phosphorylation and CCL8 expression of KCs cultured with *DMBT1*-overexpressing MCF7 CM and/or JSH-23 (30 μM). **e**–**g** CD62L induction (**e**), *Ccl8* and *Cd62l* expression (**f**) of KCs after treatment of LvM16 CM, with or without GO203, and the NET-inducing effects of the CM of such KCs (**g**). **h**–**m** GO203 treatment (15 mg/kg) of NOD-SCID mice with intrasplenic injection of MCF7 cells with *DMBT1* overexpression. Shown are flow cytometry analysis of CD62L^+^ KCs in livers (**h**), in vivo BLI (**i**), ex vivo BLI of livers (**j**), representative of BLI and metastases (**k**), NETosis in livers (**l**) and body weight changes of mice (**m**) at week 4 after cancer inoculation. *n* = 3 biological repeats (**c**, **e**–**g**), 7 (**h**, **i**, **m**), 6 (**l**) or 3 (**j**) mice. *P* values were obtained by two-tailed unpaired *t*-test (others). Scale bars, 50 μm. Data are shown as mean ± SD (others).

The mechanism of KC regulation by tumor-derived, extracellular DMBT1 was further analyzed. Previous studies have found that DMBT1 can interact with several immune defense molecules including mucin 1 (MUC1), lactoferrin (LTF), secretory IgA, surfactant protein A and D (SFTPA/SFTPD), and components of the complement system^49,52^. Among these molecules, MUC1 is a cell surface protein, and is highly expressed in KCs (Supplementary Fig. S7a). In addition, MUC1 expression is higher in the CD62L^+^ KC subcluster 6 than in other KC subclusters or macrophage/monocytes (Supplementary Fig. S7b, c). The MUC1 protein is self-cleaved into two subunits after translation, and the C-terminal subunit (MUC1-C) functions as a receptor, which can translocate into the nucleus upon ligand binding to activate NF-κB signaling by interacting with p65^53–56^. We first confirmed the interaction of DMBT1 with MUC1-C. Reciprocal co-immunoprecipitation (co-IP) assays manifested that ectopically expressed DMBT1 can interact with ectopic and endogenous MUC1-C in 293 T cells, as well as in the HL60 promyelocytes (Fig. 7a). We also observed that treating KCs with *DMBT1*-overexpressing tumor CM enhanced MUC1-C nuclear localization and NF-κB activation, while the CM of cancer cells with *DMBT1* knockdown had the opposite effects (Fig. 7b). Furthermore, *Ccl8* and *Cd62l* are both NF-κB target genes, as chromatin-immunoprecipitation (ChIP)-qPCR assays demonstrated the binding of p65 on *Ccl8* and *Cd62l* promoters (Fig. 7c). Treating KCs with the NF-κB inhibitor JSH-23 suppressed DMBT1-induced CCL8 expression and CD62L^+^ polarization (Fig. 7d; Supplementary Fig. S7d, e).

To further validate the involvement of MUC1 in regulation of KCs and metastasis, KCs were treated with GO203, a potent inhibitor of MUC1-C^57^, together with DMBT1-containing CM of LvM16 cells. GO203 treatment reduced CD62L^+^ induction (Fig. 7e), as well as *Ccl8* and *Cd62l* expression in KCs (Fig. 7f), leading to suppression of the KC capacity to activate neutrophils for NETosis in vitro (Fig. 7g). Further, when the NOD-SCID mice with intrasplenic implantation of *DMBT1*-overexpressing MCF7 cancer cells were treated by daily intraperitoneal injection of GO203, GO203 blocked the induction of CD62L^+^ KCs (Fig. 7h), reduced cancer cell colonization (Fig. 7i–k) and suppressed NET formation in the liver (Fig. 7l), although GO203 displayed considerable toxicity with significant body weight loss in treated animals (Fig. 7m).

### Therapeutic targeting of DMBT1 in liver metastasis

**Fig. 8:**
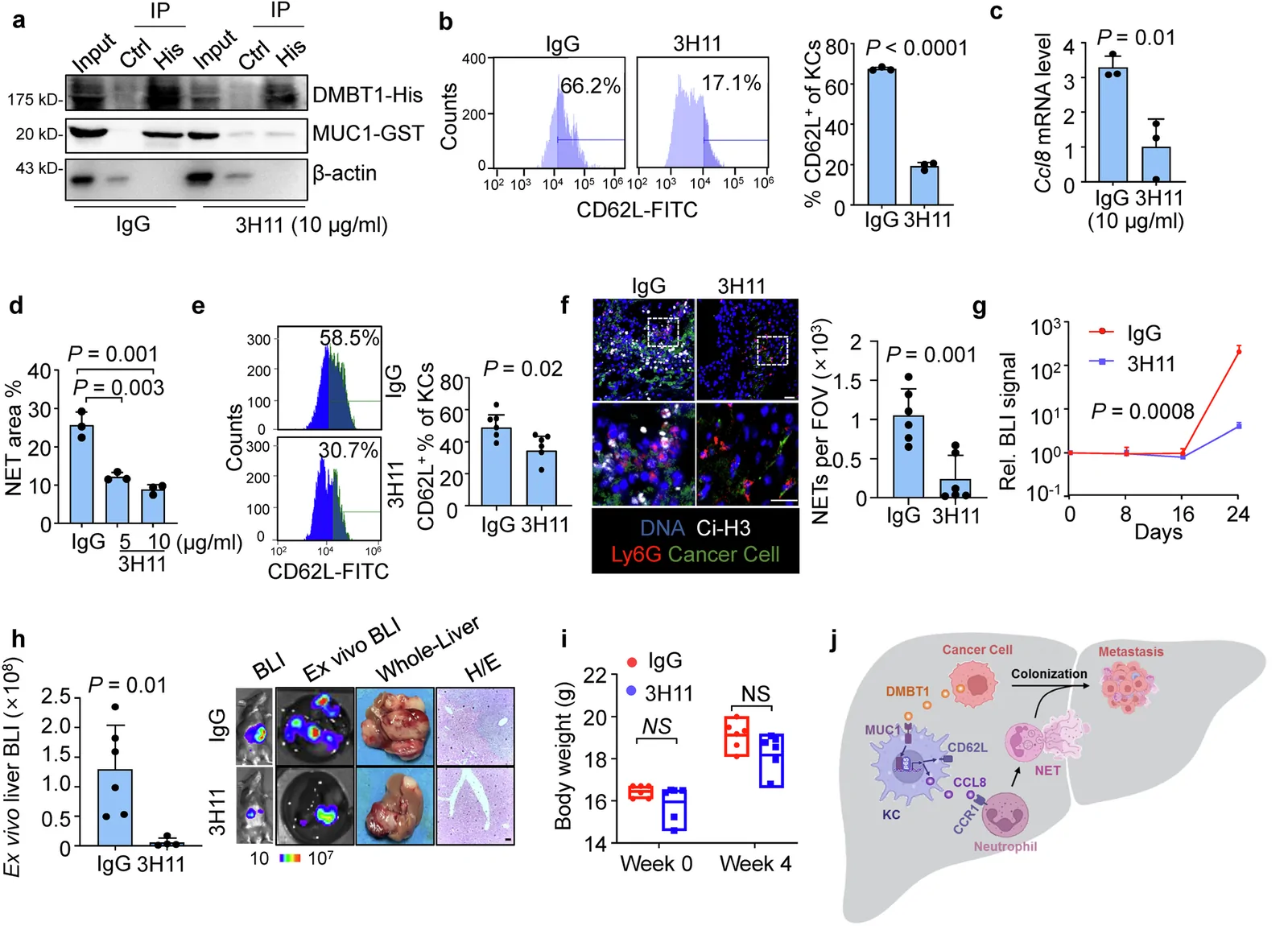
Targeting DMBT1 to treat liver metastasis. **a**–**d** In vitro analysis of the DMBT1 neutralizing antibody clone 3H11. Shown are DMBT1–MUC1 interaction in KCs after 3H11 treatment (**a**), CD62L^+^ polarization (**b**), *Ccl8* expression of KCs treated with DMBT1-containing tumoral CM with or without treatment of 3H11 (**c**) and NETosis of neutrophils after treatment of CM of the above KCs (**d**). **e**–**i** 3H11 treatment (100 μg/mouse) of C57BL/6 mice after intrasplenic inoculation of Py8119 cells. Shown are CD62L^+^ KC abundance (**e**), NETosis (**f**, zoomed areas shown at bottom) in livers, in vivo BLI (**g**), ex vivo BLI of livers and representative images of metastases (**h**), and body weight changes of the mice (**i**). **j** Schematic model of the tumor–KC–neutrophil interaction in breast cancer liver metastasis (figure created with elements from Biorender.com). *n* = 3 biological repeats (**b**–**d**) or 6 mice (**e**–**i**). *P* values were obtained by repeated measures two-way ANOVA (**g**) or two-tailed unpaired *t*-test (others). Scale bars, 50 μm. Data are shown as mean ± SEM (**g**) or mean ± SD (others).

The high expression of DMBT1 in liver-metastatic tumor cells but not in normal liver or bone marrow-derived cells^58^ indicates a strategy to inhibit tumor–KC interaction by targeting DMBT1 for metastasis treatment. Currently there are no available inhibitors of DMBT1, and thus we performed a screening for DMBT1 neutralizing antibodies and successfully identified a clone 3H11 that could detect DMBT1 (Supplementary Fig. S8a, b) and importantly, block the interaction of DMBT1 and MUC1-C (Fig. 8a). Treating KCs with 3H11 together with LvM16 CM effectively inhibited CD62L^+^ polarization and *Ccl8* expression (Fig. 8b, c). Importantly, the KCs treated with 3H11 lost the capacity to induce NETosis of neutrophils, and the effect was dose-dependent (Fig. 8d). Then, we interrogated the therapeutic potential of 3H11 in treatment of breast cancer liver metastasis. C57BL/6 immunocompetent mice were inoculated with Py8119 cancer cells by intrasplenic injection, followed by daily intraperitoneal administration of 3H11 or control IgG. 3H11 treatment reduced the CD62L^+^ KC population (Fig. 8e) and suppressed NET formation in the liver (Fig. 8f), leading to mitigation of metastatic growth of cancer and alleviation of metastatic burden (Fig. 8g, h). Importantly, 3H11 suppressed liver metastasis without obvious side effects on the body weight of the mice (Fig. 8i). The potential toxicity of 3H11 was further assessed in tumor-free C57 mice. With a 30-day continuous intraperitoneal administration of 3H11, the treatment had no apparent effects on body weight, white blood cells or major organs of the mice as compared to vehicle and IgG treatment (Supplementary Fig. S8c–f). Thus, our data manifested the potential of the DMBT1 neutralizing antibody to treat liver-metastatic breast cancer.

## Discussion

The liver is a frequent target of metastasis for many cancer types. The mechanisms of liver tropism for gastrointestinal cancers, such as gastric cancer, pancreatic cancer and colorectal cancer, have been well studied. However, the regulation of metastatic colonization of breast cancer in the liver is much less clear, although hepatic involvement is a deadly complication. Here we report the role of a distinctive subpopulation of KCs, designated by CD62L and CCL8 expression, in liver metastasis of breast cancer. These KCs are induced by *DMBT1*-expressing tumor cells, and foster metastatic growth of tumor cells by CCL8-mediated activation of NETosis. Thus, our data unravel a molecular network of tumor-niche interaction that involves both tissue-resident and bone marrow-derived immune components in the liver (Fig. 8j). The study also provided potential strategies to treat metastatic cancer, as both DMBT1 targeting with a neutralizing antibody and MUC1 inhibition by GO203 effectively suppresses NET formation and liver metastasis. An obvious side effect was observed after GO203 treatment, likely due to the expression and roles of MUC1 in the respiratory and digestive systems. The DMBT1 neutralizing antibody appeared more promising for metastasis treatment with less toxicity observed in the tested preclinical models. However, potential side effects could not be ruled out for DMBT1 neutralization, as DMBT1 also plays crucial roles in mucosal immunity. Drug delivery optimization for liver or KC targeting would be desirable to improve the therapeutic potential. In addition, it is also of interest to further analyze the mechanisms of DMBT1 upregulation in liver-tropic breast cancer, as this could provide more opportunities to specifically inhibit tumor-derived DMBT1.

NETosis is a special form of cell death for neutrophils and has been observed in multiple organs bearing cancer cells. Previously, Yang et al. noticed more NETs in the liver as compared to other tissues^18^. In this study, we confirmed this observation with clinical samples of breast cancer as well as murine tumors, validating the enrichment of NETs in liver tumors. Due to the well-established metastasis-promoting roles of NETs and the available NET-targeting strategies, this observation might have important implications for metastasis treatment. As the first line of defense, neutrophils routinely patrol liver sinusoids, but few reside in the liver. In case of acute liver infection and injury, neutrophils are rapidly recruited into the liver. Therefore, abundant neutrophil infiltration into the liver upon cancer cell seeding would be imaginable. However, it is unknown why neutrophils are activated for NETosis particularly in the liver. Our data demonstrated that the tissue-resident macrophages are mediators of tumor–neutrophil communication. Notably, the metastatic tumor cells could also directly induce NETosis, but the tumor-educated KCs are much more potent for NET induction (Fig. 4a), providing an explanation why NETs are induced in many types of metastases, but particularly abundant in liver metastases. The specific expression of MUC1 and CCL8 in KCs, but not in monocytes or monocyte-derived macrophages (Supplementary Figs. S4d, S7b, c) underlies the unique roles of KCs in response to tumoral education and in turning into metastasis accomplice.

Monocyte-derived macrophages are well known for their complicated roles in cancer, while tissue-resident macrophages have recently been a focus of study in various diseases. We identified the CD62L-expressing KC subcluster that is specifically induced by liver-tropic cancer cells. Previously Aizarani et al. also found a subcluster of KCs expressing CD62L in humans^59^, but the contribution of this KC population to liver homeostasis and diseases is unknown. Here, we showed the involvement of these KCs in driving NETosis. These findings would help the understanding of KC heterogeneity and their roles in cancer metastasis. Notably, in our study CD62L is a cell surface marker of the tumor-associated KC population. We found that CD62L did not interact with DMBT1, or affect the KC capacity to induce NETosis (data not shown). Thus, CD62L might not be directly involved in KC interaction with tumor cells or neutrophils. However, CD62L (also known as L-selectin) is a crucial transmembrane protein in circulating leukocytes and T cells, contributing to adhesion, migration and signal transduction^35,60^. Therefore, the CD62L^+^ KCs might play additional roles in cancer and liver diseases aside from NETosis induction, which are to be further investigated.

## Materials and methods

### Constructs and reagents

Human *DMBT1* was cloned into pLVX-puro-His vector for overexpression and Co-IP assays. *MUC1* was cloned into pLVX-puro-GST vector. The annealed sense and antisense shRNA oligonucleotides were cloned into pLKO.1-puro vector (Addgene) for knockdown of human *DMBT1* or murine *Dmbt1*, with the following target sequences: 5ʹ-ACCTTGAGGTTGGTCAATTTA-3ʹ (sh*DMBT1*#1), 5ʹ-CCAGGCAAATATTTACATCTT-3ʹ (sh*DMBT1*#2), 5ʹ-GTTGACTACAACTCCTTATTT-3ʹ (sh*Dmbt1*#1), 5ʹ- GGCTGCAACTATGACTATATC-3ʹ (sh*Dmbt1*#2). The antibodies used for western blotting and IF were as follows: GAPDH (G9545, Sigma), β-actin (A2228, Sigma), DMBT1 (27069-1-AP, Proteintech), Ly6G (551459, BD Pharmingen), MUC1-C (ab109185, ABCAM), p-Erk1/2 (4370, CST), Erk1/2 (9102, CST), GST-Tag (2625, CST), His-Tag (12698, CST), acetyl-histone H3 (06-599, Merck), CD62L (244495, ABCAM), p65 (sc-372, Santa Cruz), p-p65 (3033, CST), polyclonal GFP antibody (ab6673, ABCAM), CD68 (213363, ABCAM), Ci-H3 (ab5103, ABCAM), MPO (AF3667, R&D), Pan-CK (sc-8018, Santa Cruz), CLEC4F (PA5-47396, R&D), CD45 beads (130-052-301, Milteny), Debris Removal Solution (130-109-398, Milteny), Alexa Fluor 488 donkey anti-rabbit IgG (A-21206, Invitrogen), Alexa Fluor 555 donkey anti- mouse IgG (A31570, Invitrogen), Alexa Fluor 555 Donkey anti-goat IgG (A21432, Invitrogen), Alexa Fluor 647 goat anti-mouse IgG (405322, Invitrogen), HRP-conjugated goat anti-mouse IgG (401215, Merck/Millipore), HRP-conjugated goat anti-rabbit IgG (401315, Merck /Millipore). Antibodies used for FACS analyses of liver stromal components in metastases are as follows: PE-Ly6G (49-9668-80, eBioscience), APC-CD11b (17-0112-81, eBioscience), PE-F4/80 (12-4801-82, eBioscience), FITC-CD62L (104405, Biolegend).

The recombinant proteins, neutralizing antibody and reagents used in vitro and in vivo assays are human DMBT1 (11678-H08H, SinoBiological), murine CCL8 (AF790-SP, R&D), SAA1 (CSB-EP020656M0a0, CUSABIO), SAA3 (CSB-YP361411M, CUSABIO), GO203 (1222186-26-6, MCE), DNase I (4536282001, ROCHE), anti-mouse Ly6G (BE0075-1, BioXcell), GSK484 (HY-100514, MCE), estradiol valerate (S3149, Selleck), JSH-23 (749886-87-1, MCE) and DMBT1 ELISA kits (EH1756, FineTest).

### Cell lines

MDA-MB-231, LvM16, Py8119 and AT3 were grown in DMEM (11965118, Gibco) with 10% FBS, (SR100180.03, Sunrise) and 100 μg/mL penicillin/streptomycin/fungizone (15240062, Gibco). MCF7 was grown in DMEM with 10% FBS, 100 μg/mL penicillin/streptomycin/fungizone, NEAA (11140050, Gibco) and Na pyruvate (11360070, Gibco). HL60 and murine primary KCs were grown in RPMI-1640 (11875119, ThermoFisher Scientific) with 10% FBS, and 100 μg/mL penicillin/streptomycin/fungizone.

### Cytoplasm/nucleus separation

The Nucleoprotein Extraction Kit (Sangon Biotech,C500009) was used for separating cytoplasm/nuclear components of cells. Briefly, 1 × 10^6^–10^7^ cells were resuspended in 1000 μL pre-cooled Extraction Buffer 1 with 10 μL protease inhibitor and were lysed on ice for 10 min. Then the supernatant was obtained after centrifugation (3000 rpm, 10 min) as the cytoplasmic component. The precipitate was resuspended by 250 μL Extraction Buffer 3 with 2.5 μL protease inhibitor and was lysed on ice for 30 min. Then the supernatant was obtained after centrifugation (9000 rpm, 10 min) as the nuclear component.

### DMBT1 neutralizing antibody production and purification

DMBT1 neutralizing monoclonal antibody was developed by Abclonal Technology. Briefly, mice were immunized with the peptide including SRCR, CUB and SID domains of DMBT1. Hybridoma were cultured with HybGro media (H630KJ, BasalMedia) supplied with CellTurbo supplement (H460JV, BasalMedia). For neutralizing antibody production, female BALB/c mice were injected with 300 μL pristane (P9622, Sigma). 2 × 10^6^ hybridoma cells were injected intraperitoneally 7 days after pristane injection. After 7–14 days, mice were sacrificed and ascites were collected. Antibody purification was performed with Protein G resin (C600991, Sangon Biotech). Purified antibodies were dialyzed into PBS and concentrated to 1 mg/mL for in vitro and in vivo use.

### Mouse experiments

Female immunocompetent and athymic nude mice of BALB/c and C57BL/6 strains, and NOD/SCID mice aged 6–10 weeks were used in all animal experiments. C57BL/6-nude mice were obtained from the National Rodent and Leporid Laboratory Animal Resource Center of China. Orthotropic, intraperitoneal (i.p.) and intrasplenic injection were performed as previously described^61,62^. Briefly, 1 × 10^5^ cancer cells were injected into the fourth mammary fat pad for orthotopic inoculation. 1.5 × 10^6^ human cancer cells or 5 × 10^3^ murine cancer cells were injected into the spleen for liver metastasis. For murine cancer cell xenografting, the spleen was removed after intrasplenic inoculation to avoid tumor growth at the injection site. For assays with MCF7 inoculation, the mice were subcutaneously injected with 3.75 mg/kg estradiol valerate every other day. The anti-Ly6G antibody (1A8, Bio X Cell) or rat IgG at a dose of 10 μg per mouse was administered daily through i.p. administration for 20 days. The NET inhibitors GSK484 (20 mg/kg) or DNase I (5 mg/kg) was administered via daily i.p. injection for 20 days. The MUC1 inhibitor GO203 (15 mg/kg) was administered via i.p. injection every two days^57^. Clod (200 μL/mouse) were i.p. injected every 3 days for 9 days, starting from 1 day before cancer cell injection. For DMBT1 neutralizing treatment, IgG (100 μg per mouse; YEASEN, 36111ES60) or the purified 3H11 antibody (100 μg per mouse) was i.p. injected daily until the mice were sacrificed. BLI was acquired with a NightOWL II LB983 Imaging System (Berthold) and IVIS Spectrum CT (PerkinElmer). All animal studies were conducted according to the guidelines for the care and use of laboratory animals and were approved by the Institutional Animal Care and Use Committee of Shanghai Institute of Nutrition and Health.

### Preparation of liver single cell suspension

Briefly, livers were perfused with 50 mL of PBS through the hepatic portal vein or spleen until they turned white. Then the metastasis foci on liver were picked. Total livers were minced into pieces of 1–1.5 mm, further digested by 5 mg/mL Collagenase Type III (LS004182, Worthington), 0.001% (W/V) DNase 1 (D-4527, Sigma) and 1% (w/w) Dispase (17105-041, Invitrogen) at 37 °C for 20–40 min. Then, 5 mL blocking buffer was added to stop the liver digestion. The suspension was filtered through a 100 μm strainer and 15 mL blocking buffer was used to rinse the strainer. The resulting cells were centrifuged at 1500 rpm for 8 min at 4 °C. The supernatant was gently removed. Finally, erythrocytes were lysed in ice-cold RBC lysis buffer (555899, D) for 5 min.

### RNA-seq and scRNA-seq analyses

RNA-seq of neutrophils was performed as previously described^63^. Briefly, total RNA of neutrophils was extracted using the Trizol reagent (15596018, Invitrogen). Library construction and sequencing were performed at WuXi NextCODE, Shanghai. Upregulated and downregulated genes with fold changes > 2 and Benjamini-Hochberg adjusted *P* values < 0.05 were identified.

For scRNA-seq analysis, two mice with MDA-MB-231 or LvM16 inoculation were sacrificed 7 days after intrasplenic xenografting and single cell suspension of liver metastasis was prepared as described above. The cells were sorted with CD45 mouse microbeads (5211005943, Miltenyi Biotec) to enrich CD45^+^ immune cells. Library construction and sequencing were performed at Oebiotech, Shanghai. Briefly, the CD45^+^ cells were loaded on a Chromium Single cell Instrument with Chromium Next GEM Single Cell 3ʹ Reagent Kits v3.1 (10× Genomics) to generate single cell GEMs. After quality control by fragment analysis (AATI), libraries were sequenced by the Illumina sequencer. The sequencing data were processed with the Cell Ranger pipeline (V.2.0.1; 10× Genomics) using default settings. Cells were included for further analysis if the number of expressed genes was greater than 200 and the mitochondrial gene expression ratio was less than 5%. Gene expression matrix was normalized using log-norm and the data were used for subsequent clustering analysis, differential expression and Principal component analysis.

### KC isolation

The single cells prepared from murine livers as described above were laid on top of a 2-layer Percoll (17089102, GE Healthcare) gradient (50% and 25% in sterile Hank’s buffered salt solution (HBSS)), followed by centrifugation at 1200× *g* for 30 min at 25 °C. KCs enriched in the interface fraction were confirmed to be of > 80% purity by flow cytometric analysis (Supplementary Fig. S3e).

### Neutrophil isolation and analysis

For mouse neutrophil isolation, bone marrow cells were harvested from 6 to 10-week-old BALB/c mice in HBSS without Ca^2+^/Mg^2+^ (14185052, Invitrogen), and laid on top of a 2-layer Percoll gradient (72% and 65% in HBSS), followed by centrifugation at 1200× *g* for 30 min at 25 °C. Neutrophils enriched in the interface fraction were confirmed to be of > 95% purity by flow cytometric analysis (Supplementary Fig. S4a). Neutrophils were cultured with 1640 medium.

For NETosis analysis, 2.5 × 10^5^ neutrophils were seeded on coverslips coated with poly-L-lysine (P4707, Sigma) in 24-well plates for 30 min before adding 50% cancer cell CM, G0-203 (10 μM) or PMA (20 nM, Sigma). After 12 to 16 h culturing at 37 °C, neutrophils were fixed with 4% paraformaldehyde for 10 min at room temperature, washed twice with pre-chilled PBS, permeabilized in 0.1% Triton X-100 for 5 min, and then washed twice with PBS. Cells were blocked in PBS containing 5% BSA for 30 min, then incubated with anti-Ly6G (1:100, 551459, BD Pharmingen) and Ci-H3 (1:200, ab5103, ABCAM) in blocking buffer overnight at 4 °C. After three washes in PBS, cells were incubated with fluorochrome-conjugated secondary antibodies (1:500, Invitrogen) for 1 h, then counterstained with mounting medium with DAPI (ab104139, ABCAM). Observation and photographing were performed with the confocal microscopy Cell Observer (ZEISS, Germany). Image processing and analysis were performed with Zen blue edition software (ZEISS, Germany). For NET quantitation of in vitro culture, the MPO/Ly6G^+^Ci-H3^+^ and DAPI-stained areas on the slides were quantified by the ImageJ software, and NET area% was measured as MPO/Ly6G^+^Ci-H3^+^ area/DAPI^+^ area. For NET quantitation of in vivo tissue samples and clinical samples, the numbers of MPO/Ly6G^+^ Ci-H3^+^ spots were counted by ImageJ in every field of view.

For neutrophil recruitment analysis, 5 × 10^5^ freshly isolated murine neutrophils were added to the upper chamber (363096, BD), and rCCL8 in the RPMI1640 medium was added to the lower chamber as the chemoattractant. The migrated cells in the lower chamber were counted after 3 h.

### Western blotting

Cultured cells were harvested using a scraper or via trypsinization, and then lysed in the lysis buffer (50 mM Tris-HCl, 150 mM NaCl, 1% NP-40 detergent, 0.5% sodium deoxycholate, 0.1% SDS with phosphatase and protease inhibitors) on ice for 15 min. The lysates were subsequently centrifuged at 13,300 rpm for 15 min. The resulting supernatants were boiled at 95 °C for 10 min. Finally, the samples were subjected to SDS-polyacrylamide gel electrophoresis. The blots were quantified using ImageJ software and measured as the normalized intensity ratios of target proteins to loading control (β-actin or GAPDH).

### ChIP

ChIP assays for p65 binding of *Ccl8* and *Cd62l* promoters were conducted in 293 T cells. Briefly, 293 T cells were crosslinked with 1% formaldehyde and quenched by 125 mM glycine. Cell nuclear lysate was sonicated and incubated with control IgG or anti-p65 antibody for immunoprecipitation. The complex was captured and precipitated by agarose beads. Captured genomic DNA was reverse-crosslinked and purified by ethanol precipitation with MinElute purification kit (28004, Qiagen). Purified genomic DNA was used for qPCR analysis.

### Immunoprecipitation

Cells were collected and then lysed with immunoprecipitation buffer (150 mM NaCl, 20 mM HEPES pH 7.4, 1% Triton X-100, 12.5 mM β-glycerophosphate, 1.5 mM MgCl_2_, 2 mM EGTA) with inhibitors (10 mM NaF, 1 mM PMSF, 1 mM Na_3_VO_4_ and Protease inhibitor cocktail). Equal amounts of protein were incubated with the primary antibodies or control antibodies overnight at 4 °C, followed by incubation with protein A or protein G dynabeads (GE Life Sciences) for 2 h at 4 °C. The samples were washed 3 times with immunoprecipitation buffer before being resolved by SDS-PAGE and immunoblotted with the indicated antibodies. For Ni-NTA bead immunoprecipitation, cells were lysed with lysis buffer (50 mM NaH_2_PO_4_, 300 mM NaCl, 10 mM imidazole, pH 8.0) and equal amounts of protein were incubated with Ni-NTA beads (20502ES10, YEASEN) overnight at 4 °C, followed by washing with the wash buffer (50 mM NaH_2_PO_4_, 300 mM NaCl, 20 mM imidazole pH 8.0) and elution with the elution buffer (50 mM NaH_2_PO_4_, 300 mM NaCl, 250 mM imidazole, pH 6.2) for 20 min at room temperature. The supernatant was boiled for 10 min at 95 °C before being resolved by SDS-PAGE and immunoblotted with the indicated antibodies.

### Clinical analysis

In the Qilu breast cancer cohort, human paraffin-embedded specimens, including primary tumors, LN, liver, lung and bone metastases, and serum were obtained from Qilu Hospital of Shandong University, with informed consent from all subjects and approval from the Institutional Research Ethics Committee of Qilu Hospital (reference no. KYLL-2016-350). Serum samples were analyzed with the DMBT1 ELISA kit.

The UNC breast cancer cohort was previously published^46,47^ and the datasets (GSE3521, GSE26338) were obtained from the NCBI GEO database. The primary tumor samples with clinical information and available expression data of *NUCB2* and *DMBT1* were analyzed. Samples were split by *NUCB2* or *DMBT1* mRNA levels into the high-expression (higher one third) and low-expression (lower two thirds) groups, and patient survival was compared.

### Statistical analyses

Data analyses were performed using GraphPad Prism 8.0 (GraphPad Software, La Jolla, USA). The data presentation and statistical analyses are described in the figure legends. *P* < 0.05 were considered statistically significant. The in vitro experiments were independently repeated multiple times with similar results, as indicated in the figure legends.

## Supplementary information

Supplementary Figures and Tables

## Data Availability

The data of secretomic profiling, RNA-seq and scRNA-seq were deposited in the National Omics Data Encyclopedia (NODE) and can be accessed with the accession code OEP00005641 (https://www.biosino.org/node/project/detail/OEP00005641). Original data of all figures were deposited in the Mendeley Database (https://data.mendeley.com/drafts/px5ymz9wkn). All other data of this study are available from the corresponding author upon reasonable request.
