# Author Correction: Breast cancer induces CD62L^+^ Kupffer cells via DMBT1 to promote neutrophil extracellular trap formation and liver metastasis

**DOI:** 10.1038/s41421-026-00932-2

**Published:** 2026-10-13

**Authors:** Pu Tian, Qiuyao Wu, Dasa He, Wenjing Zhao, Lichao Luo, Zhenchang Jia, Wenqian Luo, Xianzhe Lv, Yanan Liu, Yuan Wang, Qian Wang, Peiyuan Zhang, Yajun Liang, Qifeng Yang, Guohong Hu

**Affiliations:** 1 Shanghai Institute of Nutrition and Health, University of Chinese Academy of Sciences, Chinese Academy of Sciences, Shanghai, China; 2 Department of Breast Surgery, Qilu Hospital of Shandong University, Ji’nan, Shangdong China

Correction to: *Cell Discovery* https://www.nature.com/articles/s41421-025-00819-8, published online 12 August 2025

In Fig. 6g of the initially published version of the article, the representative images of KD1 and KD2 contained partially overlapped/similar areas due to a mislabeling for the file name, and thus a KD1 (kd4 in file names) image was mislabeled as KD2 (kd5 in file names), or vice versa. In addition, the *P* value was mislabeled from 0.0003 to 0.003. The corrected Fig. 6 is provided below, with both mislabeled images replaced and *P* value corrected. The authors apologize for the negligence and any inconvenience caused by the erratum. The figure error did not affect any conclusion of the article.

**Correct Fig. 6**

**Fig. 6:**
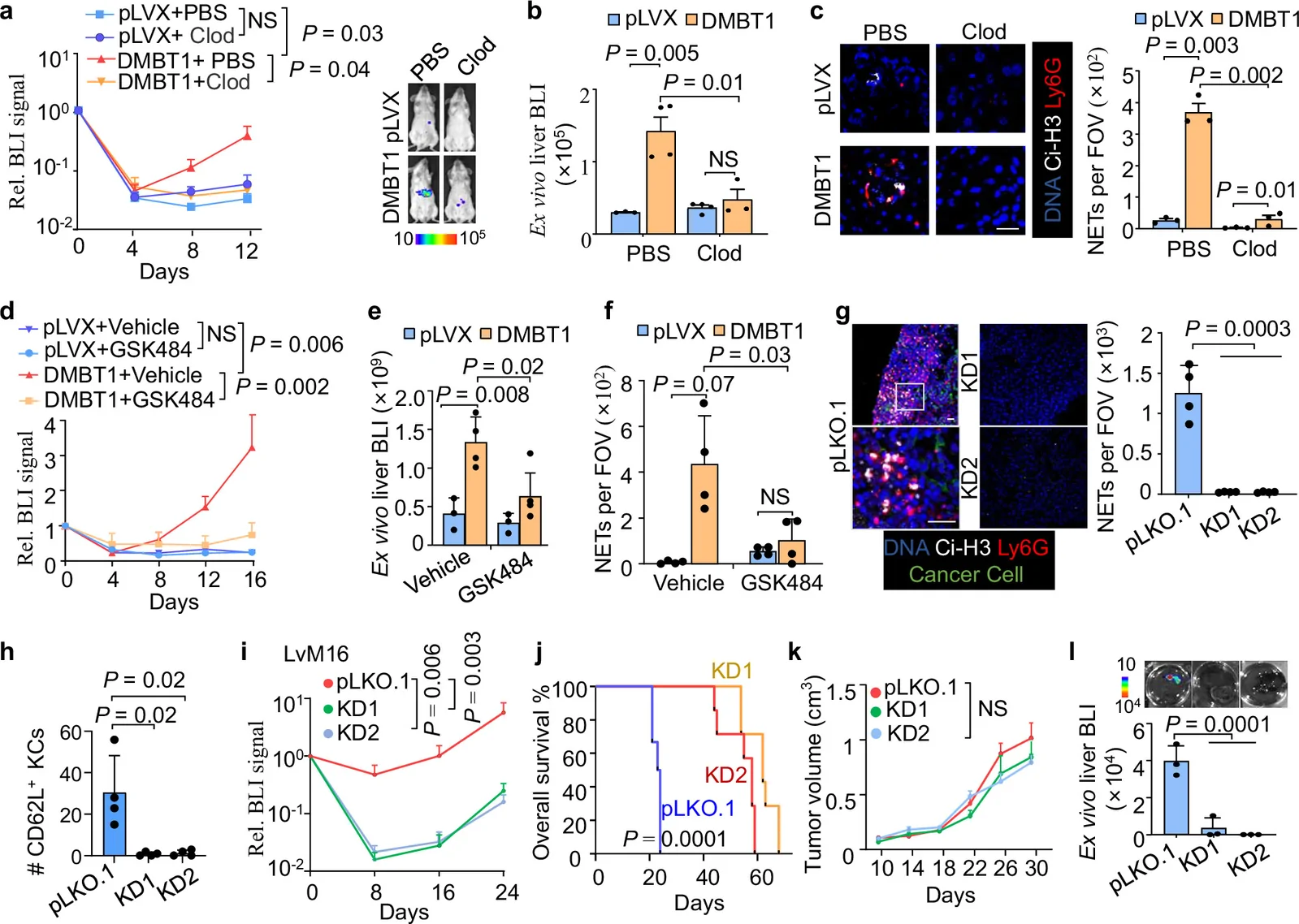
DMBT1 promotes liver metastasis of breast cancer. **a**–**c** In vivo BLI (**a**), ex vivo BLI (**b**) and NETosis (**c**) in livers of NOD-SCID mice treated with Clod after intrasplenic inoculation of MCF7 with or without *DMBT1* overexpression. **d**–**f** In vivo BLI (**d**), ex vivo BLI (**e**) and NETosis (**f**) of NOD-SCID mice treated with GSK484 after intrasplenic inoculation of MCF7 with or without *DMBT1* overexpression. **g**–**j** NETosis (**g**), CD62L^+^ KCs (**h**) and in vivo BLI (**i**) of livers, and survival (**j**) of nude mice after intrasplenic injection of LvM16 cells with *DMBT1* knockdown. **k**, **l** Primary tumor growth (**k**) and liver metastasis (**l**) in C57BL/6 mice after orthotopic injection of AT3 cells with *Dmbt1* knockdown. *n* = 3 or 4 (**a**, **b**), 3 (**c**, **h**, **k**, **l**), 7 (**d**, **i**, **j**), 4 (**e**, **f**, **g**) mice per group. *P* values were obtained by repeated measures two-way ANOVA (**a**, **d**, **i**, **k**), Log rank test (**j**) or two-tailed unpaired *t*-test (others). Scale bars, 50 μm. Data are shown as mean ± SEM (**a**, **d**, **i**, **k**) or mean ± SD (others).

**Incorrect Fig. 6**

**Fig. 6:**
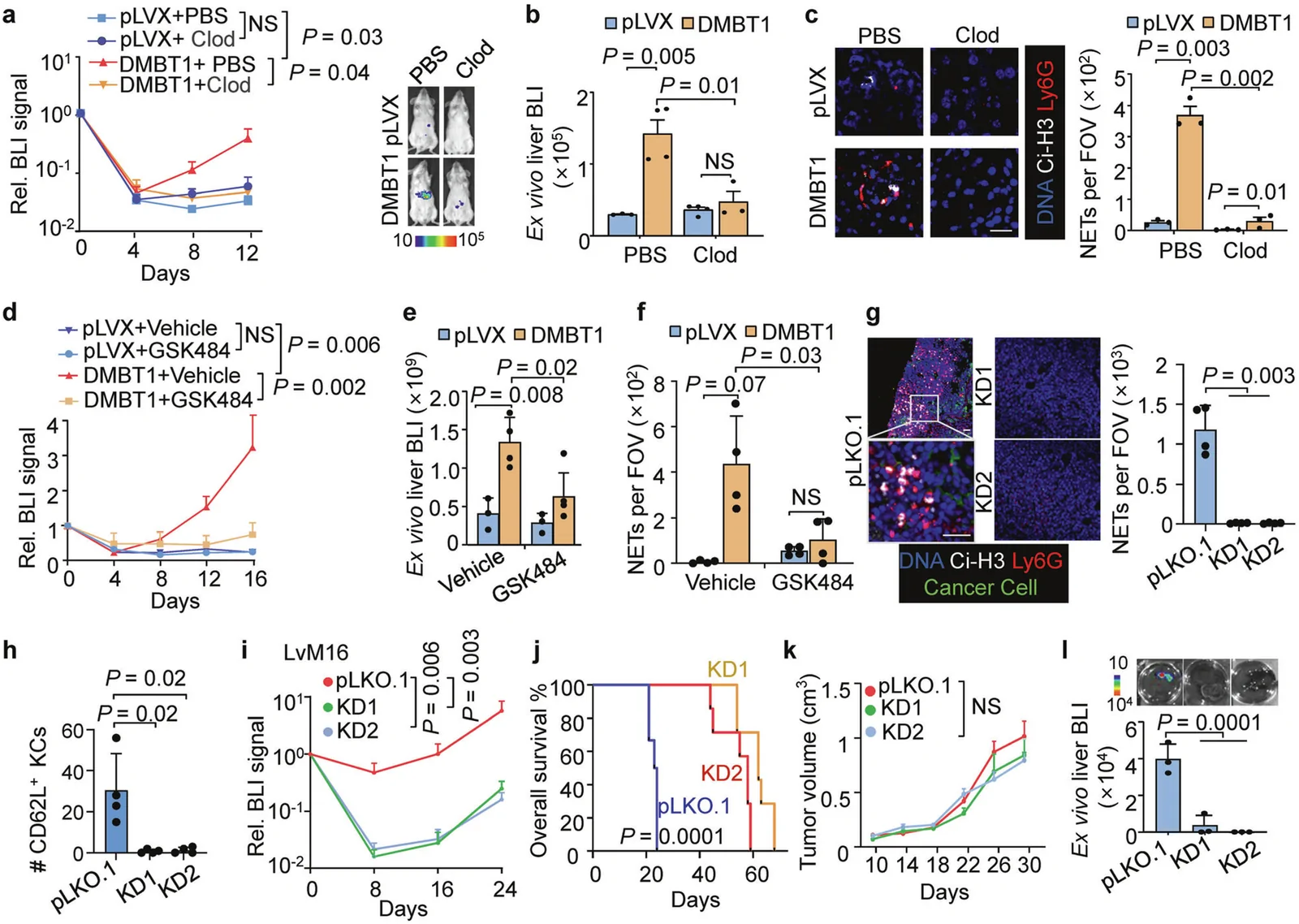
DMBT1 promotes liver metastasis of breast cancer. **a**–**c** In vivo BLI (**a**), ex vivo BLI (**b**) and NETosis (**c**) in livers of NOD-SCID mice treated with Clod after intrasplenic inoculation of MCF7 with or without *DMBT1* overexpression. **d**–**f** In vivo BLI (**d**), ex vivo BLI (**e**) and NETosis (**f**) of NOD-SCID mice treated with GSK484 after intrasplenic inoculation of MCF7 with or without *DMBT1* overexpression. **g**–**j** NETosis (**g**), CD62L^+^ KCs (**h**) and in vivo BLI (**i**) of livers, and survival (**j**) of nude mice after intrasplenic injection of LvM16 cells with *DMBT1* knockdown. **k**, **l** Primary tumor growth (**k**) and liver metastasis (**l**) in C57BL/6 mice after orthotopic injection of AT3 cells with *Dmbt1* knockdown. *n* = 3 or 4 (**a**, **b**), 3 (**c**, **h**, **k**, **l**), 7 (**d**, **i**, **j**), 4 (**e**, **f**, **g**) mice per group. *P* values were obtained by repeated measures two-way ANOVA (**a**, **d**, **i**, **k**), Log rank test (**j**) or two-tailed unpaired *t*-test (others). Scale bars, 50 μm. Data are shown as mean ± SEM (**a**, **d**, **i**, **k**) or mean ± SD (others).

