# Supplementary material for: Breast cancer induces CD62L+ Kupffer cells via DMBT1 to promote neutrophil extracellular trap formation and liver metastasis
Source: Cell Discov. 2025 Aug 12;11:68. doi: 10.1038/s41421-025-00819-8 (PMC12343785; doi:10.1038/s41421-025-00819-8)
Supplement: Supplementary file 1 — Supplementary Figures and Tables [file 41421_2025_819_MOESM1_ESM.pdf]

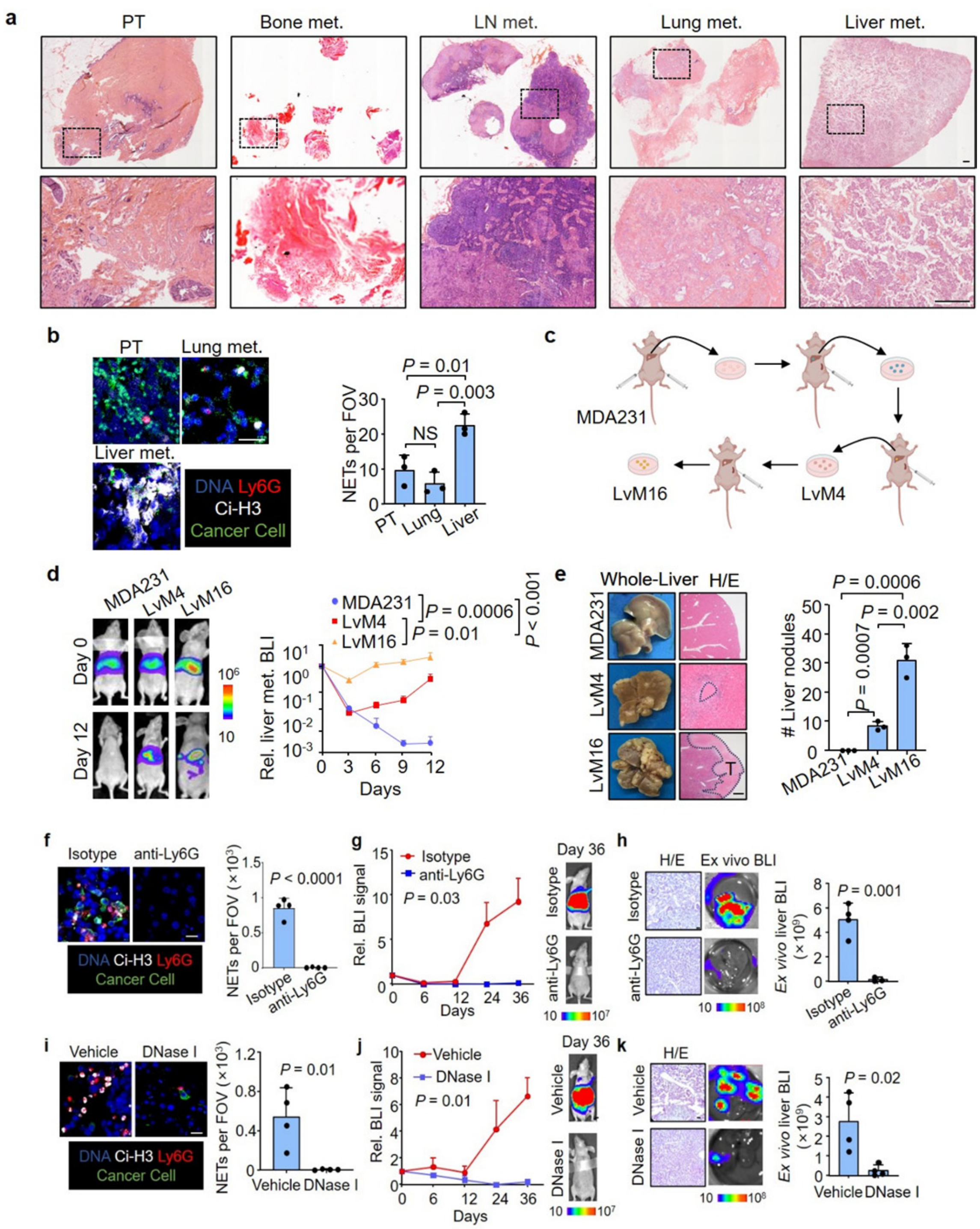

Fig. S1 Analysis of NETs in breast cancer metastasis.

a Representative images of hematoxylin & eosin (H&E) staining of primary tumors (PT) and metastases (met.) of human breast cancer. Areas in box are enlarged at bottom. **b** NET abundance in primary tumors (on day 30 after orthotopic injection), lung (on day 10 after intravenous injection) and liver metastasis (on day 10 after intrasplenic injection) of C57BL/6 mice with Py8119 cancer cells. **c** Schematic of experimental procedure for *in vivo* selection of liver-tropic sublines of MDA-MB-231 (MDA231) cells. **d, e** Liver

metastasis capacity of parental MDA-MB-231 and two sublines (LvM4 and LvM16) in mice after intrasplenic injection. **f-h** Anti-Ly6G treatment (10 μg per mouse) of nude mice after LvM16 intrasplenic inoculation for liver metastasis analyses. Shown are IF analysis of NETs (**f**), *in vivo* BLI (**g**), H/E staining and *ex vivo* BLI of livers (**h**). **i-k** DNase I treatment (5mg/kg) of nude mice after LvM16 intrasplenic inoculation for liver metastasis analyses. Shown are IF analysis of NETs (**i**), *in vivo* BLI (**j**), and H/E staining and *ex vivo* BLI of livers (**k**). n = 3 (**b**, **d**, **e**) or 4 (**f**-**k**) mice per group. *P* values were obtained by repeated measures two-way ANOVA (**d, g, j**) or two-tailed unpaired t-test (others). NS, not significant. Scale bar, 100 μm (**a**, **e, h, k**) or 20 μm (**b**, **f, i**). Data are shown as mean ± SEM (**d, g, j**) or mean ± SD (others).

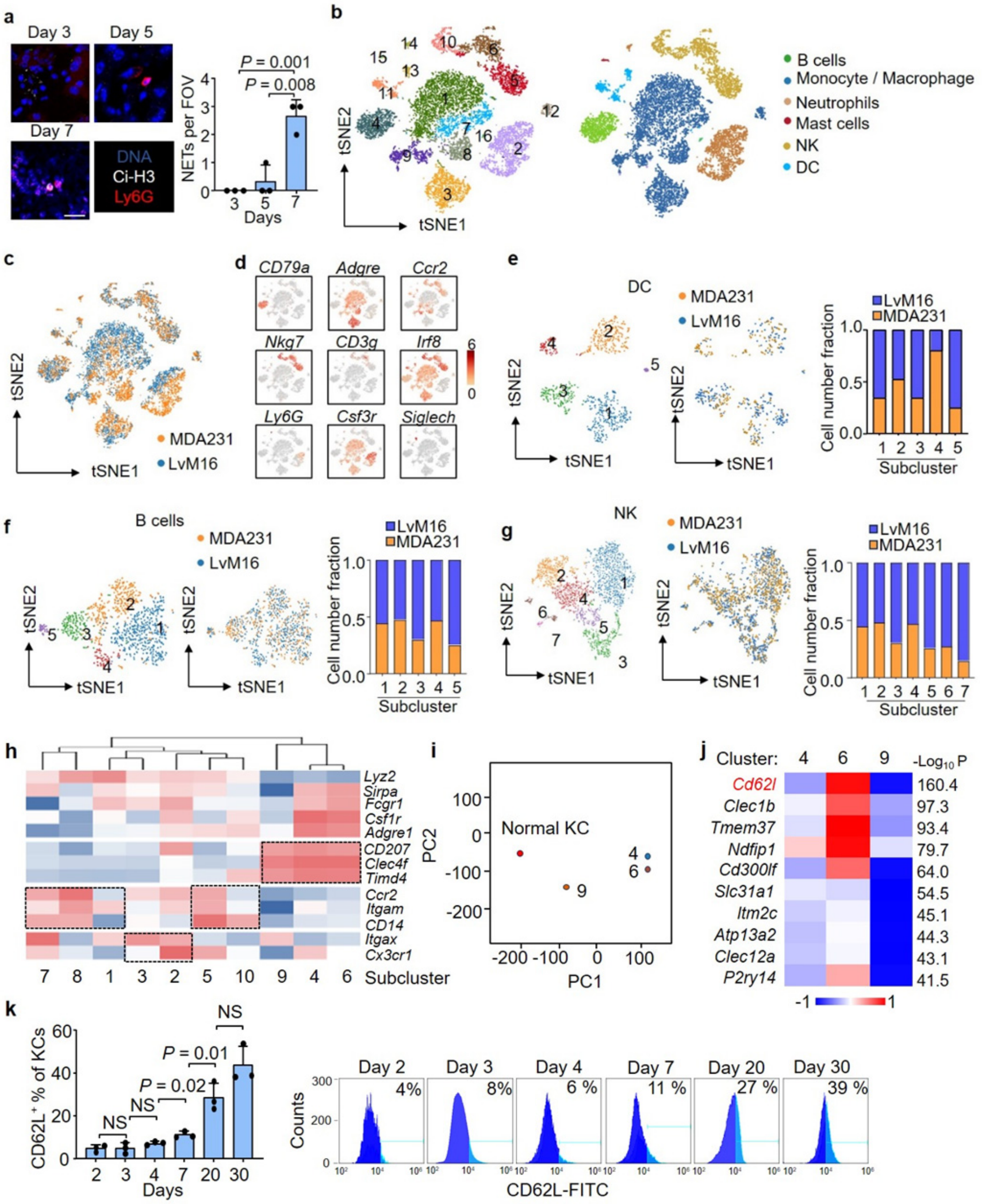

Fig. S2 Cell component changes in liver metastasis niche.

a IF analysis of livers of nude mice at different time points after LvM16 intrasplenic inoculation. **b-j** scRNA-seq analyses of CD45$^+$ cells in livers on day 7 after MDA-MB-231 (MDA231) or LvM16 intrasplenic inoculation. Shown are tSNE distribution of CD45$^+$ cells according to cell cluster and types (**d**), tSNE distribution and abundance of subclusters of DC (**e**), B cells (**f**) and NK cells (**g**) in MDA231 and LvM16-inoculated livers, heatmap visualization of marker gene expression in different macrophage subclusters (**h**), scRNA-seq principal-component analysis (PCA) of macrophage subclusters 4, 6 and 9 identified in tumor-bearing livers and normal KCs isolated from tumor-free mice (**i**; scRNA-seq data of normal KCs were derived from a separate assay in

the laboratory), heatmap visualization of differentially expressed cell surface protein-encoding in KCs subclusters (**j**, *P* values were obtained by two-tailed unpaired t-test of subcluster 6 versus others). **k** Flow cytometry analysis of $CD62L^+$ KC abundance in livers at different time points after LvM16 inoculation. n = 3 mice (**a**, **k**) per group. *P* values were obtained by two-tailed unpaired t-test. Data are shown as mean ± SD.

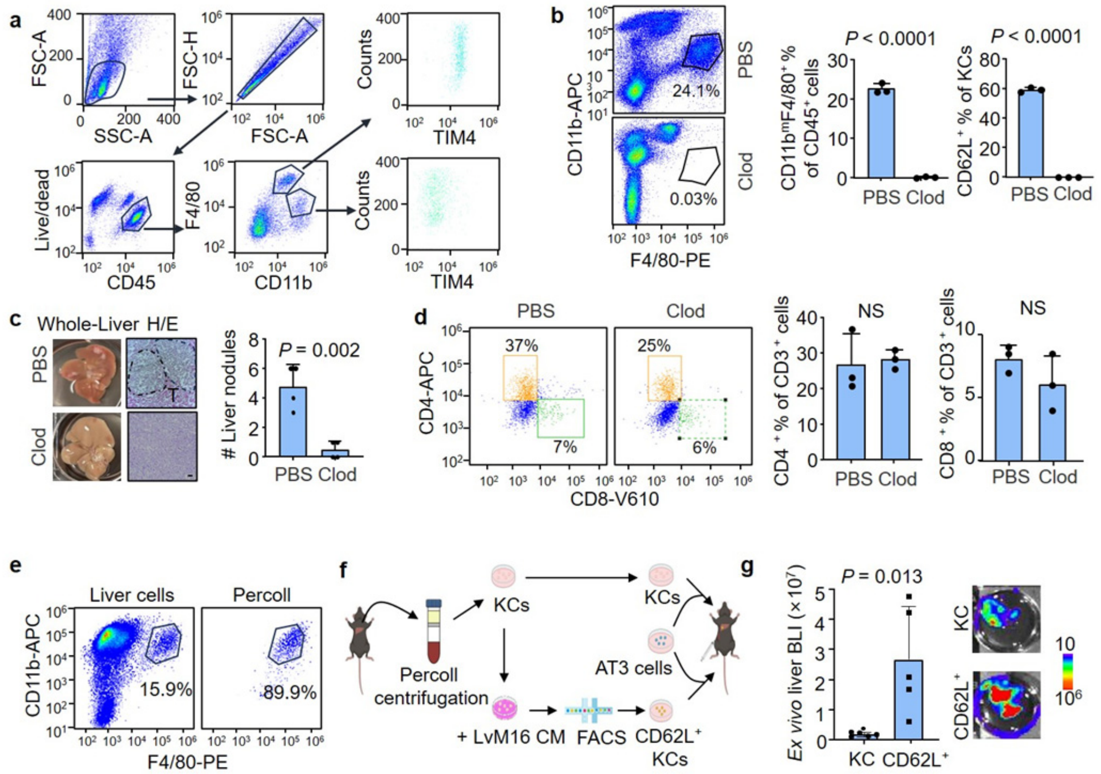

Fig. S3 CD62L$^+$ KCs promote liver metastasis.

a Flow cytometry gating strategy of KCs. CD11b^{m}F4/80$^+$ cells were identified as KCs and TIM4 expression in both CD11b^{m}F4/80$^+$ and CD11b$^+$F4/80^m were then analyzed. **b-d** The effect of clodronate liposome (Clod) treatment of C57BL/6 mice with intrasplenic inoculation of AT3 cells. Shown are analyses of total KC and CD62L$^+$ KC abundance (**b**), liver metastatic nodules (**c**) and CD4$^+$ / CD8$^+$ T cell abundance in livers after the treatment (**d**). **e** Flow cytometry analysis to validate the purity of Percoll-isolated KCs from healthy murine livers. **f** Schematic overview of procedure to isolate and test the function of CD62L$^+$ KCs. **g** *Ex vivo* liver BLI of mice inoculated with AT3 and total KCs or CD62L$^+$ KCs. n = 3 (**b**, **d**), 4 (**c**) or 5 (**g**) mice per group. *P* values were obtained by two-tailed unpaired t-test. Data are shown as mean ± SD.

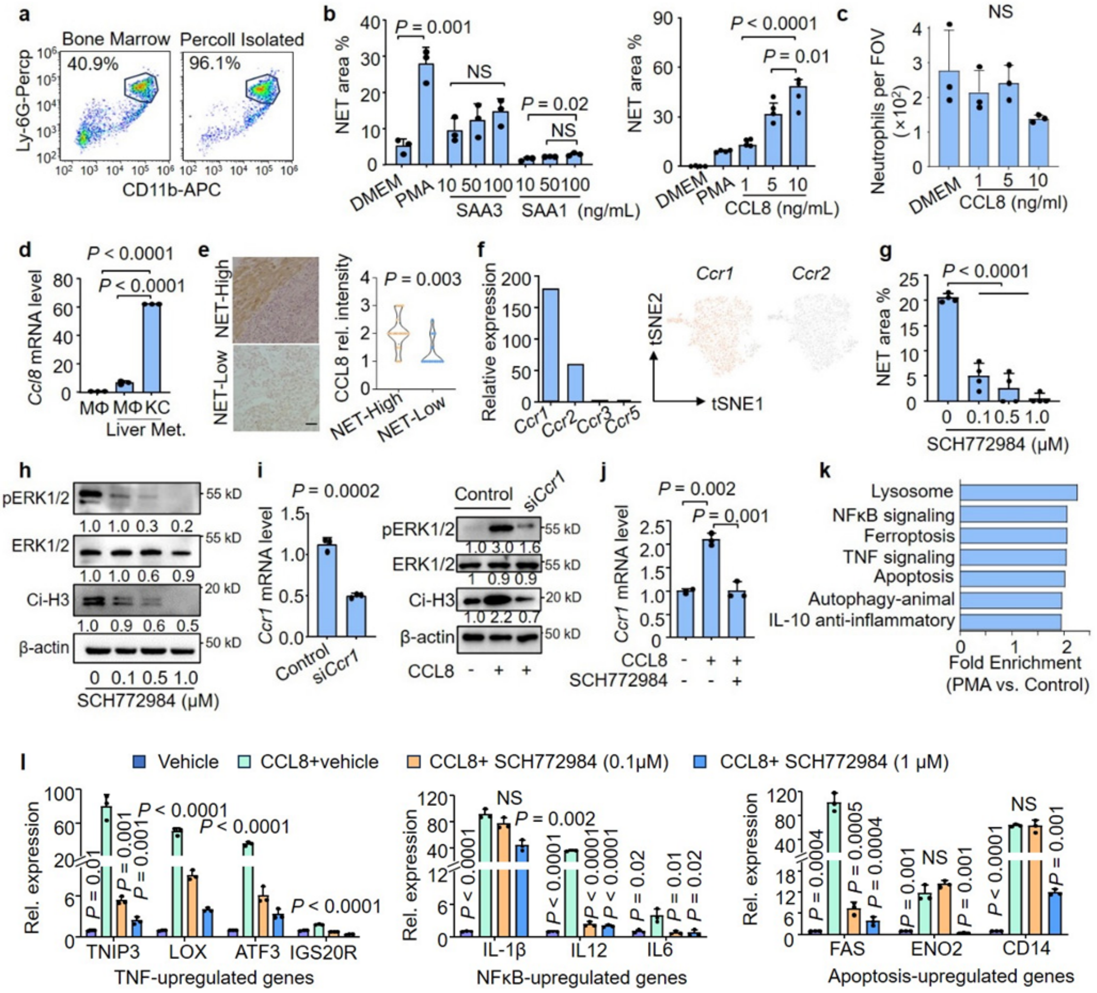

Fig. S4 CD62L^{+} KCs induce NETosis via CCL8-ERK signaling.

a Flow cytometry to validate the purity of Percoll-isolated neutrophils from bone marrow of tumor-free mice. **b** NETosis of neutrophils treated with PMA or different concentrations of recombinant SAA1, SAA3 and CCL8. **c** Neutrophil recruitment by different concentrations of recombinant CCL8. **d** *Ccl8* expression of macrophages isolated from tumor-free mice, or macrophages and KCs isolated from LvM16 liver metastases. **e** Immunohistochemistry of CCL8 in human liver metastases of breast cancer of the Qilu cohort. **f** Expression of CCL8 receptors *Ccr1*, *2*, *3* and *5* in neutrophils from tumor-free mice analyzed by RNA-seq (left) and expression of *Ccr1* and *Ccr2* in neutrophils identified in liver metastases by scRNA-seq (right). **g, h** NETosis and ERK phosphorylation of murine neutrophils treated with various concentrations of SCH772984 (SCH) for 16 h. **i** *Ccr1* expression and ERK phosphorylation of neutrophils treated with recombinant CCL8 (10 ng/mL) and/or *Ccr1* siRNA for 16 h. **j** *Ccr1* expression in murine primary neutrophils treated with CCL8 and/or SCH. **k** GO analyses of the upregulated genes in PMA-treated vs. control neutrophils. **l** qPCR analysis of the expression of genes in TNF, NFκB and apoptosis pathways in neutrophils treated with CCL8 and different concentrations of SCH. n = 9/7 patients (**e**), or 4 (**b**) or 3 (others)

biological repeats per group. *P* values were obtained by two-tailed unpaired t-test. Scale bar, 50 μm. Data are shown as mean ± SD.

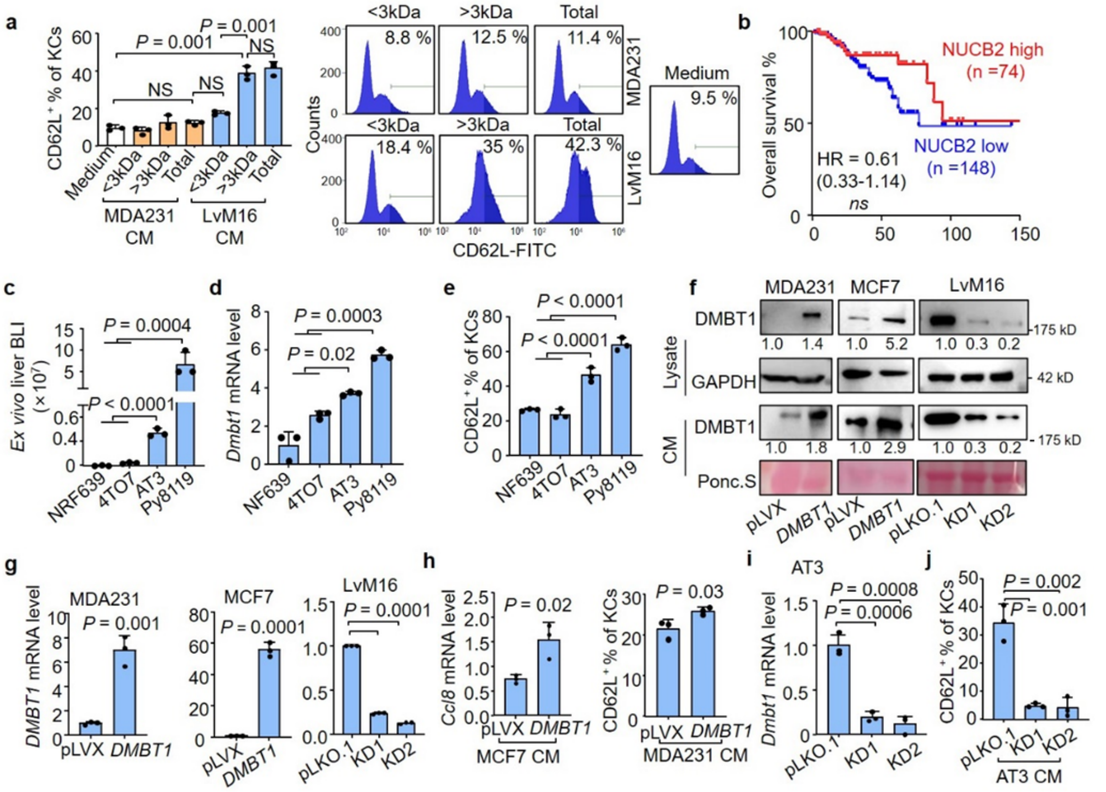

Fig. S5 DMBT1 induces CD62L$^+$ KCs.

a Flow cytometry of CD62L$^+$ KCs with control medium or different fractions of MDA-MB-231 or LvM16 CM. **b** Overall survival of breast cancer patients stratified by *NUCB2* expression in the UNC cohort (GSE3521, GSE26338). **c** Liver metastasis capacity of murine breast cancer cells. Shown are *ex vivo* liver BLI 6 weeks after intrasplenic injection of the indicated cell lines in syngeneic mice (FVB for NF639, and BALB/C for others). 2×10^5 (NF639 and 4TO7) or 5×10^3 (AT3 and Py8119) cells were injected. **d, e** *Dmbt1* mRNA expression in murine cell lines (**d**) and CD62L induction of primary KCs treated with the CM of the cell lines (**e**). **f-g** *DMBT1* mRNA and protein expression in cancer cells with *DMBT1* overexpression or knockdown. **h** *Ccl8* expression and CD62L induction of KCs treated with CM of *DMBT1*-overexpressing cancer cells. **i, j** qPCR validation of *Dmbt1* knockdown efficiency in AT3 (**i**) and CD62L induction of KCs treated with CM of *Dmbt1*-silenced AT3 (**j**). n = 222 patients (**b**) or 3 biological repeats (others). *P* values were obtained by Log-rank test (**b**) or two-tailed unpaired t-test (others). Data are shown as mean ± SD.

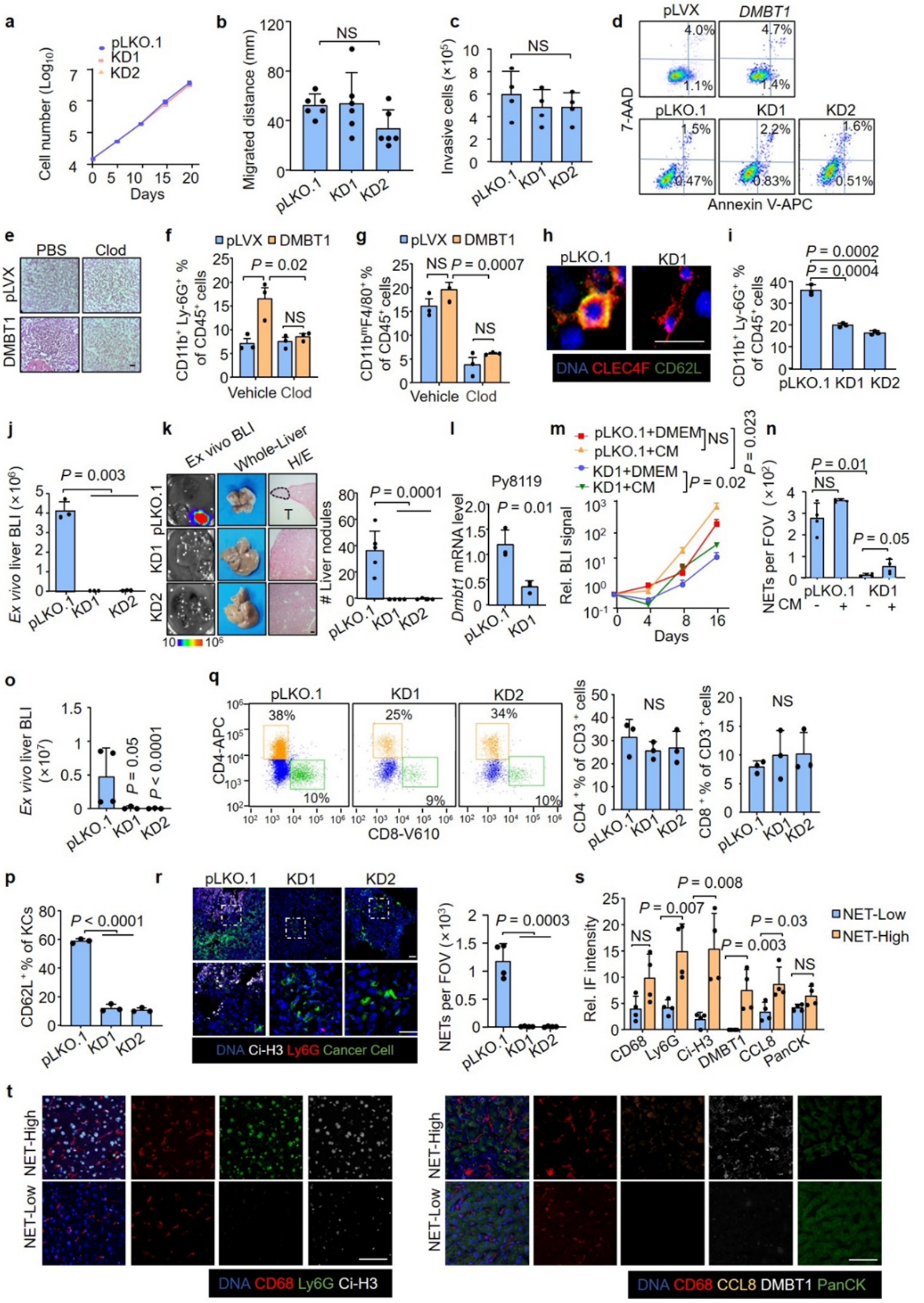

Fig. S6 The roles of DMBT1 in cancer cells and liver microenvironment.

a-d The effects of DMBT1 on *in vitro* growth (**a**), migration (**b**), invasion (**C**) and apoptosis of tumor cells (**d**). **e-g** NOD-SCID mice were treated with clodronate liposomes after intrasplenic injection of MCF7 cells with *DMBT1* overexpression for liver metastasis analysis. Shown are H&E staining (**e**), neutrophil (**f**) and KC abundance (**g**) in livers. **h-k** IF analysis of CD62L^{+} KCs

by CM of LvM16 cells with *DMBT1* knockdown (**h**), and neutrophil abundance (**i**), *ex vivo* BLI (**J**) and metastasis burden (**k**) in livers of mice after intrasplenic inoculation of these LvM16 cells. **l-n** Py8119 CM treatment of C57BL/6 mice after intrasplenic inoculation of Py8119 cells with or without *Dmbt1* knockdown for metastasis analysis. Shown are validation of *Dmbt1* knockdown in Py8119 (**l**), *in vivo* BLI (**m**) and NETosis in livers (**n**). **o-q** Intrasplenic injection of AT3 cells with *Dmbt1* knockdown for analysis of liver metastasis. Shown are *ex vivo* BLI (**o**), and abundance of CD62L$^+$ KCs (**p**) and CD4$^+$/CD8$^+$ T cells in liver (**q**). (**r**) NETosis in livers of mice with orthotopic injection of AT3 with *Dmbt1* knockdown (zoomed areas shown at bottom). **s, t** Multiplex immunofluorescence analyses for cancer cells (PanCK), KCs (CD68), CCL8, DMBT1 and NETs in liver metastases of breast cancer patients. n = 3 biological repeats (**a**-**c**), or 3 (**f**-**j**, **l, p, q**), 4 (**n, o, s**), 5 (**k, r**), 7 (**m**) mice, or 4 random microscopic fields (**s**) per group. *P* values were obtained by repeated measures two-way ANOVA (**a, m**) or two-tailed unpaired t-test (others). Scale bar, 50 μm. Data are shown as mean ± SEM (**a, m**) or mean ± SD (others).

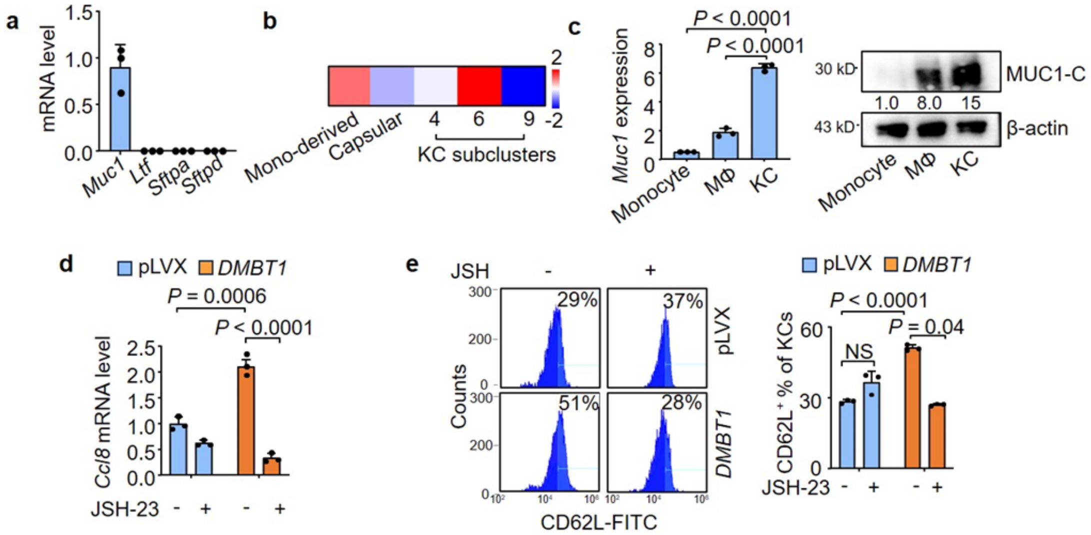

Fig. S7 KCs polarization regulated by JSH-23 via MUC1-NF-κB signaling. **a** qPCR analysis of candidate DMBT1-interacting surface proteins in KCs. **b** scRNA-seq analysis of *Muc1* expression in macrophages and KCs. **c** *Muc1* expression in monocytes, macrophages and KCs isolated from mice. **d, e** *Ccl8* expression (**d**) and $CD62L^+$ induction (**e**) of KCs cultured with *DMBT1*-overexpressing MCF7 CM and/or JSH-23 (30 μM). n = 3 biological repeats. *P* values were obtained by two-tailed unpaired t-test. Data are shown as mean ± SD.

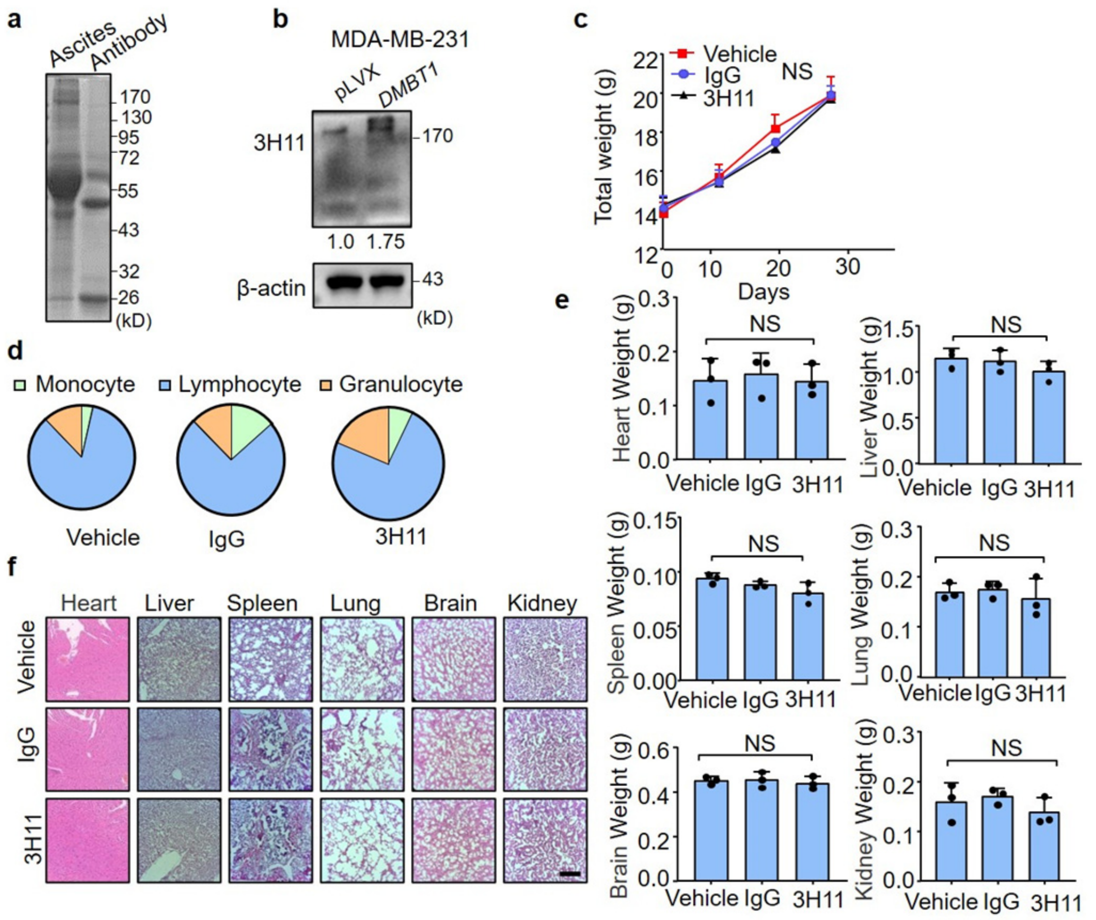

Fig. S8 Analysis of the 3H11 clone.

a Coomassie blue staining of ascites from mice inoculated with the 3H11 hybridoma and the purified 3H11 antibody from the ascites. **b** Western blotting of cell lysate of MDA-MB-231 with or without *DMBT1* overexpression using the 3H11 antibody. **c-f** Tumor-free C57BL/6 mice were treated with 3H11 (100 μg/mouse), IgG or vehicle by intraperitoneal injection. Shown are body weight changes of mice (**c**), white blood cell components (**d**), weights (**e**) and H&E staining (**f**) of main organs.

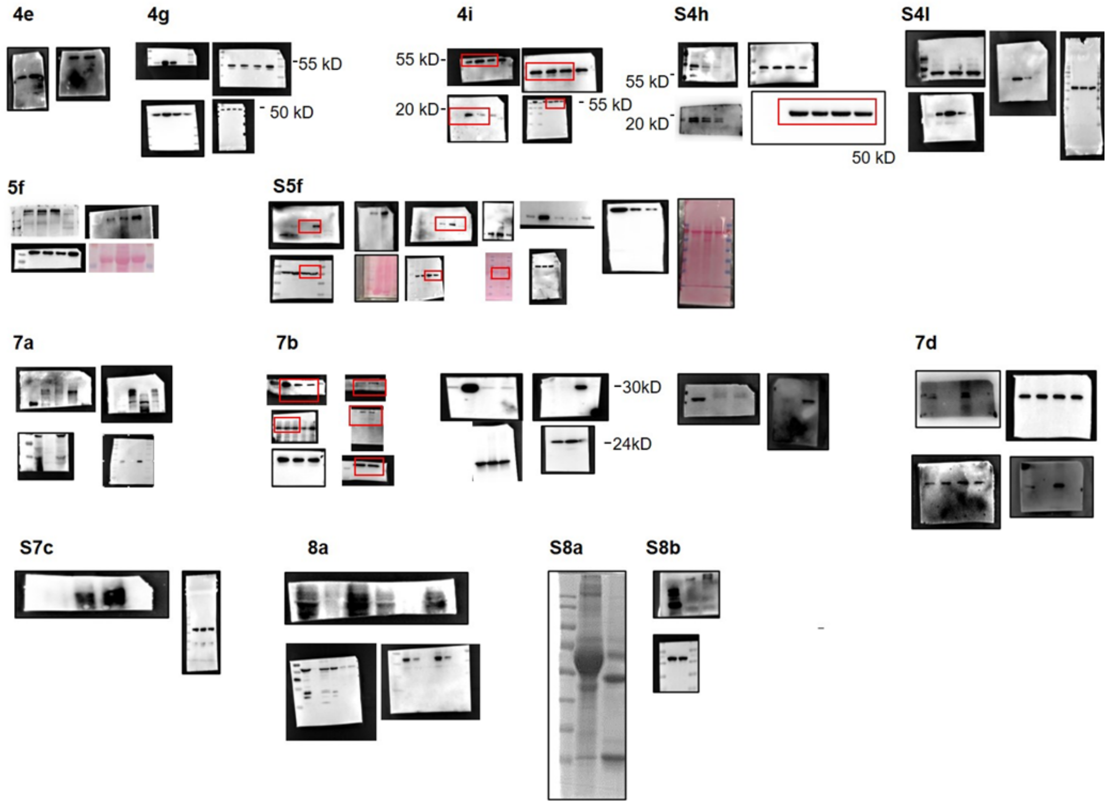

Fig. S9 Original, uncropped images of Western blots in main and supplementary figures.

Supplementary Table S1. Cell surface protein-encoding genes differentially expressed in KC subclusters

Gene	Expression fold change (subcluster 6/subclusters 4, 9)	*P* value
Cd62l	9.625	4.15E-161
Clec1b	1.052	4.89E-98
Tmem37	1.419	3.81E-94
Ndfip1	1.081	2.26E-80
Cd300lf	2.272	9.84E-65
Slc31a1	2.192	3.08E-55
Itm2c	1.083	8.41E-46
Atp13a2	1.203	5.34E-45
Clec12a	1.115	8.93E-44
P2ry14	2.991	3.17E-42
Sys1	1.345	1.10E-39
Mxra8	2.725	1.76E-39
S100a10	2.5	1.55E-36
Slc46a3	3.12	2.62E-36
Fxyd5	1.43	1.39E-35
Creld1	3.011	2.98E-35
Cd82	2.124	1.48E-33
Txndc16	2.322	3.33E-33
Trem2	1.843	1.02E-32
Wbp1	2.569	1.20E-31
Mboat7	2.156	2.99E-31
Glipr1	2.5	1.77E-30
Tmem19	2.422	2.11E-30
Colec12	1.323	3.81E-30
Ms4a7	1.865	1.46E-28
Gpr137b	1.9	1.67E-28
Tmem192	1.968	2.38E-28
Tmem175	2.492	1.55E-27
Pigp	1.843	2.22E-27
Slc39a11	2.584	2.60E-27
Atp1b3	1.223	3.31E-27
Nceh1	1.498	6.59E-27
Gbp2	2.081	1.76E-26
Fam173a	1.488	1.07E-25
Golm1	2.475	4.52E-25
Gpr35	2.233	5.83E-25
Dpy19l1	2.027	1.09E-24
Gpr146	1.966	2.38E-24
Abhd12	1.206	3.12E-24

Lpcat2	1.621	3.36E-24
Myof	1.634	3.49E-24
Tmem26	1.793	5.84E-24
Timd4	1.035	1.02E-23
Lst1	1.017	1.49E-23
Smim19	1.753	2.93E-23
Paqr4	2.145	6.74E-23
Txndc15	1.769	7.93E-23
Galnt7	2.176	9.72E-23
Atp11c	2.061	1.79E-22
Tmem131	1.752	1.04E-21
Crlf2	1.532	1.72E-21
Cd84	1.343	2.11E-21
Trpm2	1.698	2.29E-21
Ninj1	1.083	5.76E-21
Slc14a1	2.366	6.58E-21
Wdr83os	1.76	1.03E-20
Ttyh2	1.936	1.38E-20
Tmem134	1.242	1.50E-20
Slc41a3	2.192	2.03E-20
Gbp3	1.543	3.86E-20
Slc22a23	2.03	1.35E-19
Atp11b	1.952	1.65E-19
Tspan3	1.783	2.84E-19
Tmem106b	1.937	3.12E-19
Ino80c	2.12	4.64E-19
Wbp1l	1.674	1.22E-18
Gramd1a	1.854	1.28E-18
Fuca2	1.496	1.42E-18
Tmem59	1.111	1.61E-18
Heatr5a	1.899	1.78E-18
Mospd3	1.845	4.00E-18
Gramd1b	1.798	4.01E-18
Tmem63a	1.791	5.27E-18
Itfg1	1.842	8.63E-18
Slc43a2	1.288	9.37E-18
Grina	1.306	9.76E-18
Tmbim1	1.687	1.49E-17
Cd37	1.701	2.07E-17
Smim8	2.053	2.30E-17
Pianp	1.479	3.31E-17
Slc48a1	1.248	5.02E-17
Snx14	2.09	5.06E-17
St6galnac4	1.772	6.93E-17

Lancl1	2.198	7.69E-17
Relt	2.155	8.04E-17
Ilvbl	1.938	1.36E-16
Tmem88	2.097	2.02E-16
Smim12	1.857	2.76E-16
Tmem60	1.882	2.99E-16
Emd	1.688	1.34E-15
Alox5ap	1.411	1.83E-15
Tmem222	1.811	2.93E-15
Cyb561d2	1.912	4.32E-15
Ccdc167	2.067	4.69E-15
Cd47	1.043	5.73E-15
B3galnt1	1.954	6.04E-15
Dennd5b	1.77	7.42E-15
Ttyh3	1.492	7.92E-15
Tmem251	1.629	8.19E-15
Cmtm4	1.925	1.52E-14
